# The Omics Molecule Extractor: A Web Application for the Selection of Potential Biomarker Panels

**DOI:** 10.1021/acs.jproteome.5c00176

**Published:** 2025-12-01

**Authors:** Emanuel Lange, Kay Schallert, Johannes Schwerdt, Susmita Ghosh, Andreas Hentschel, Yvonne Reinders, Robert Heyer

**Affiliations:** 1 Leibniz-Institut für Analytische Wissenschaften - ISAS - e.V., Dortmund 44139, Germany; 2 Graduate School Digital Infrastructure for the Life Sciences, Bielefeld University, Bielefeld 33615, Germany; 3 38907 Hochschule Merseburg, Merseburg 06217, Germany; 4 Multidimensional Omics Data Analysis, Faculty of Technology, Bielefeld University, Bielefeld 33615, Germany

**Keywords:** feature selection, patient classification, biomarkers, machine learning, multiomics

## Abstract

Selecting molecular panels that are applicable to classify the health state of patients is a common task in omics data analysis. Existing software for molecule selection lacks features to select molecule panels from large data sets, requires programming experience, or lacks user-friendly interfaces. We present the Omics Molecule Extractor (OMEx), an open-source web application providing a user-friendly workflow for selecting molecules and molecule panels for sample classification from large data sets. OMEx’s user interface provides interactive visualization for exploring input data and analysis results. The feature selection strategy underlying the algorithm is based on machine learning and has not been available in any software with a user interface. Extensive testing using synthetic data sets with known ground truth showed that the algorithm discovers group-separating molecules with high precision. Additionally, OMEx was tested on five real-world omics data sets, demonstrating high reproducibility and overlap with reported molecules from other feature selection methods, while also reporting alternative molecules of interest. OMEx is freely available at https://mdoa-tools.bi.denbi.de/omex/home.

## Graphical abstract

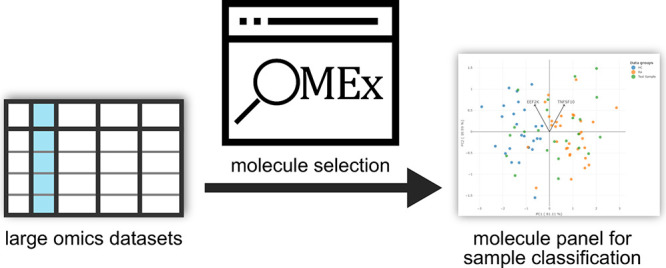

## Introduction

Omics technologies aid in investigating qualitative and quantitative presence of biomolecules (i.e., DNA, RNA, proteins, lipids, and metabolites) in biological systems, thereby enhancing the mechanistic understanding of those molecules in health and pathophysiological states.^1^ Omics methods employ cutting-edge technologies like genome sequencing and liquid chromatography coupled to mass spectrometry, generating raw data (e.g., reads and mass spectra), which are further processed to identify and quantify the biomolecules.^1^ This initial step typically generates tables containing thousands of molecules in rows, samples in columns, and measured quantities in each cell.

A common objective of applying omics technologies is to identify a subset of molecules showing quantitative differences between states of an investigated biological system, for example, between healthy and pathophysiological states. These molecules can provide insights into disease-specific processes and could serve as biomarkers for diagnosis and prognosis or as therapeutic targets.

In essence, biomarker applications, such as prognosis or diagnosis, are classification tasks that predict a sample’s class based on molecular quantities. Combining multiple molecules into panels is preferred for classification, as it typically achieves higher accuracy than relying on individual molecules. The identification of molecules and molecule panels used for classification, a process known as “feature selection”, is facilitated by various statistical and machine learning methods.^2,3^

The software implementing these methods is often only available as libraries for programming languages.^4,5^ Some of these programming libraries offer high flexibility but low user-friendliness. In contrast, software implemented as a web application can provide higher user-friendliness due to a graphical user interface accessed through web browsers, but can have limited features. Existing web applications like MetaboAnalyst^6^ or CombiRoc^7^ can generate rankings for individual molecules or molecule panels based on a limited number of input molecules, respectively. However, to our knowledge, no web application currently offers the capability to generate molecule panels from thousands of input molecules, which is the typical size of omics data sets.

Here, we present the Omics Molecule Extractor (OMEx; version 0.2.0), a user-friendly web application for the selection of molecular panels tailored to researchers who generate omics data. A unique feature of OMEx is its ability to generate panels of fewer than ten molecules that can be experimentally validated, unlike other selection methods that only produce ranked lists of candidate molecules based on criteria such as p-values. Additionally, it offers a tidy user interface and interactive visualizations, making it a compelling alternative to existing tools for molecule selection.

## Implementation

The initial version of OMEx’s algorithm was developed to determine biomarkers from a metaproteomics data set.^8^ Based on this initial version, we extended and generalized the algorithm to be applicable to other types of omics data.

### Description of the Input files

The input for OMEx is a data table obtained from omics-specific processing of raw data. The input format of this data table is a tab-separated .csv, .tsv, or .txt file containing molecule names in rows, sample names in columns, and measured quantities in cells. Sample column names are prefixed with condition names (e.g., “control_” and “disease_”) for grouping.

### Description of OMEx’s Algorithm

OMEx’s algorithm combines statistical and machine learning methods (Figure 1). The main objective is to determine a small subset of molecules from the input table, i.e., a molecule panel, which can discriminate between samples from two different conditions (e.g., healthy vs disease). The algorithm involves four steps, i.e., (1) data preprocessing, (2) (pre)­filtering of molecules based on *p*-values, (3) (post)­filtering by a wrapper, and (4) a final classification. During data preprocessing (step 1), samples can be normalized by the sum of all molecule quantities within each sample, and molecules with sparse measurements (i.e., few measured values) can be filtered out. Both operations can be disabled if users have already applied preprocessing to their data.

**Figure 1:**
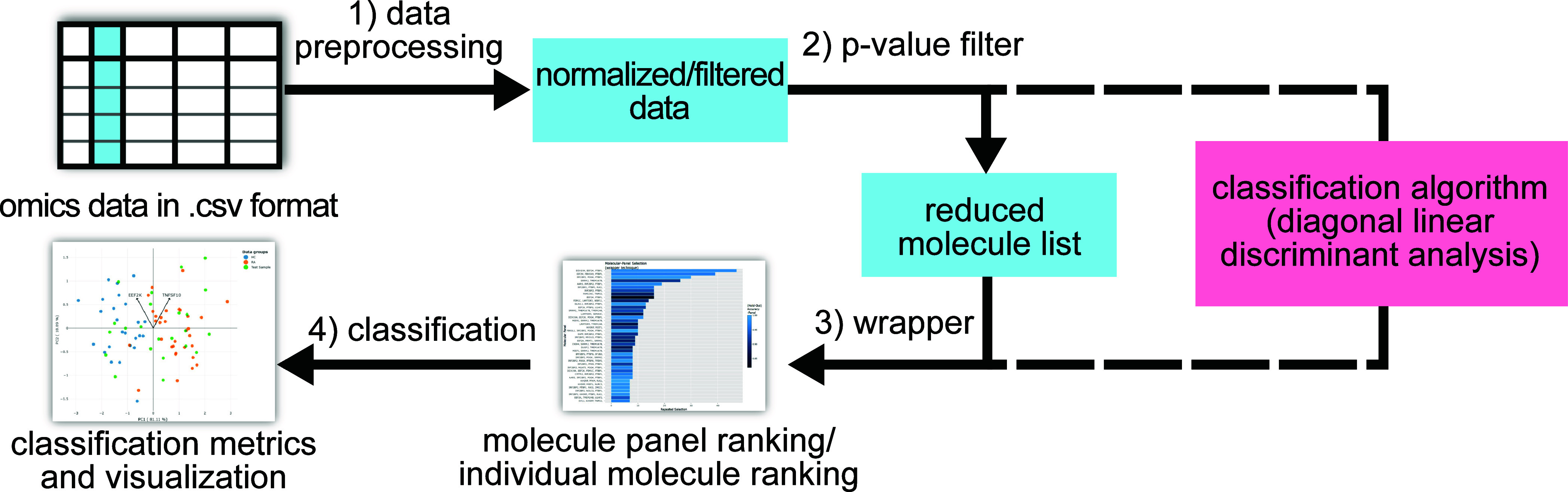
Overview on OMEx’s algorithm. The algorithm consists of an optional data preprocessing step (step 1), molecule selection via filter (step 2) and wrapper (step 3), and sample classification (step 4).

The remaining steps utilize diagonal Linear Discriminant Analysis (d-LDA),^9^ a simple classification method, and cross-validation. D-LDA has been chosen as a classification method because it is computationally lightweight and showed the best classification accuracy for a metaproteomics data set in a comparison with other classification methods.^8^

The *p*-value filter (step 2) performs a statistical test (two-sample *t* test) on each molecule independently and ranks them by their *p*-values.^2^ Molecules below a certain *p*-value cutoff are provided for the next step. The *p*-value cutoff is chosen by the algorithm to provide an optimal trade-off between classifier accuracy and a low number of molecules. In step 3, the wrapper method^2^ selects a small panel of molecules for a subset of samples and evaluates the classification accuracy based on the selected panel (cross-validation). This process is repeated several times (>1000 times), while the samples provided for molecule selection are randomized in every repetition, varying the composition of the selected panels. The wrapper outputs molecule panels and individual molecules that are ranked based on their frequency of selection. An advantage of the wrapper over p-value filtering is that combinations of molecules are considered in the classification. However, wrapping is a computationally expensive technique; Therefore, the preceding *p*-value filtering reduces the total computation time.

The most frequently chosen panels are assumed to be robust discriminators between the sample groups and are provided to the final classification step 4. Step 4 evaluates the predictive power of the selected molecule panel based on classification metrics (accuracy, precision, recall, f1 score). Additionally, principal component analysis (PCA) and hierarchical clustering are performed to visualize the separation of groups and the similarity of samples based on the selected molecule panel.

OMEx is an open-source web application. Its frontend is implemented in Angular 18 and available at https://gitlab.com/mdoa-group/mpa-website. The backend is written in Java 17 for a REST API and implementation of the algorithm, utilizes R 4.3.2 for generating plots, and uses Docker for deployment. The backend code is available at mdoa-group/mpa-cloud-server. Additionally, OMEx can be installed locally using Docker Desktop (Supporting Information, Section 9).

## Results and Discussion

### OMEx Provides a User-Friendly Interface

OMEx is available at https://mdoa-tools.bi.denbi.de/omex/home and provides a user-friendly interface based on the step-by-step workflow of the algorithm. Initially, users provide their omics data table in a tab-separated format, with molecules as row names and samples as columns (example data sets are available on the OMEx Web site and in the Supporting Information). The initial input form allows for filtering sparsely measured molecules and sample-wise normalization. The web application handles data sets with a minimum of 15 samples per group, but more samples are recommended. Users can choose to split samples into an independent test set for evaluating the predictive power of the extracted molecules. However, this is recommended only for sufficiently large data sets (e.g., >1000 samples), as removing samples from smaller data sets can increase variability in the selected molecule panels across repeated runs. Alternatively, for smaller data sets (<1000 samples), repeated cross-validation can be used. While this approach may slightly overestimate performance metrics, it typically results in more stable molecule selection.

Each workflow step contains a detailed description and generates interactive plots for analysis of its results, such as an initial overview of class balances (Figure 2A) or a ranking of selected molecule panels (Figure 2B). Parameters of the algorithm, such as cross-validation folds, can also be configured in each step, but an automatic mode running all steps at once with default parameters is available as well. In the final step, a classification of the test set based on a selected molecule panel is performed and can be evaluated using a volcano plot (Figure 2C), a pairwise scatter plot (Figure 2D), a PCA plot (Figure 2E), and important metrics for classification (accuracy, precision, recall, and f1 score). All results can be downloaded as a .zip directory, which contains all figures and a configuration file storing all settings for reproducibility. OMEx’s analyses may take several hours to complete, depending on the data set size. To accommodate this, submitted jobs can be revisited using a job ID, and users can opt to receive email notifications upon completion.

**Figure 2:**
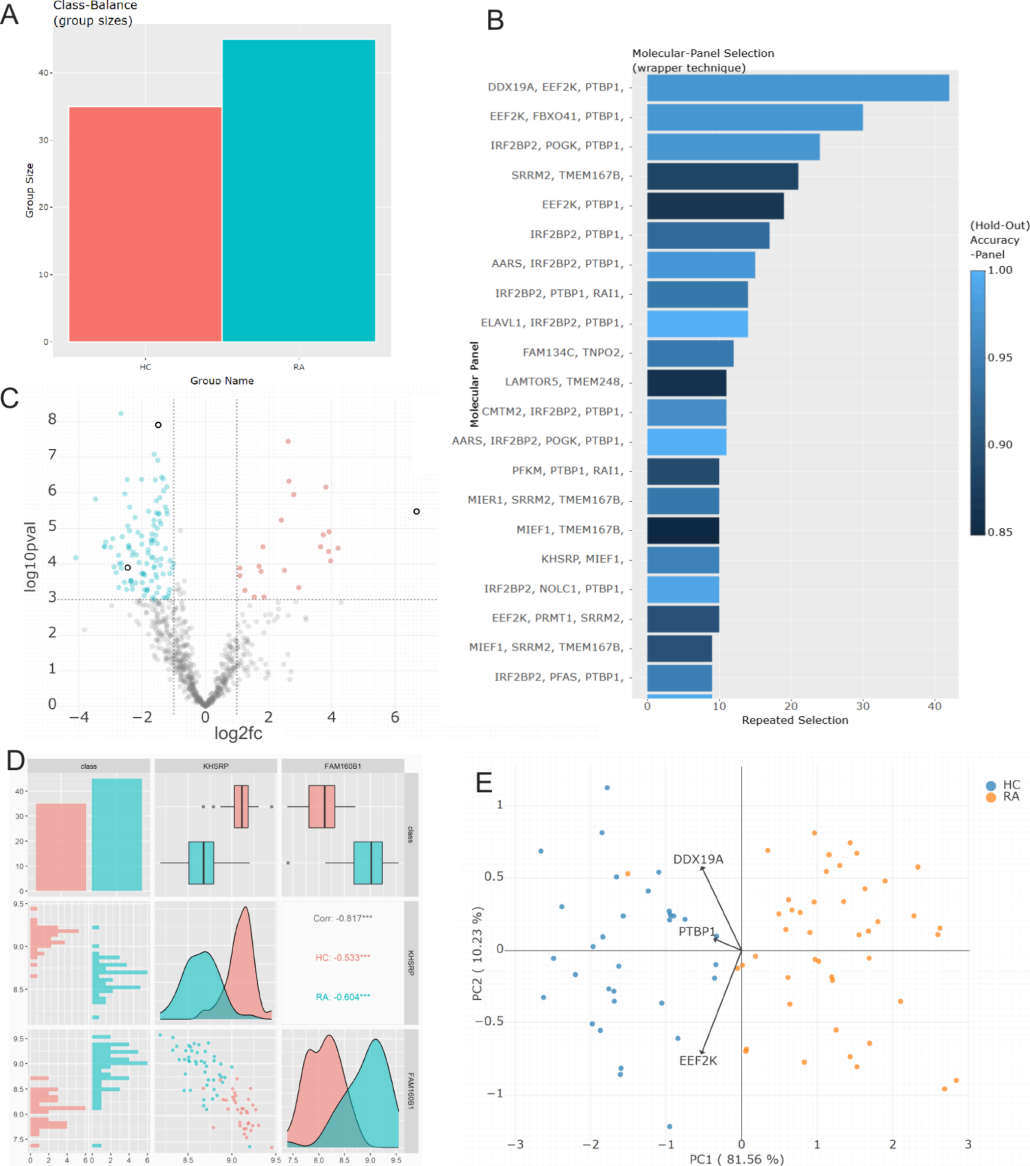
Overview of OMEx’s visualizations. The web-based user interface provides interactive plots on class balances (A), most frequently selected molecule panels (B), volcano plots visualizing differences of extracted molecules between classes (C), and pairwise scatter plots based on extracted molecules to assess class separation (D), and a PCA plot (E).

### Synthetic Data Demonstrates OMEx’s High Precision in Selecting Relevant Molecules

For a proof-of-concept, a first test of the algorithm was performed using synthetic data sets generated by a sampling strategy. The filter and wrapper were tested individually and in combination (Table 1) by applying 100 synthetic data sets, respectively. All synthetic data sets contained 60 samples and 50 “synthetic molecules” (a detailed description of the sampling strategy can be found in the Supporting Information. The code is implemented in Java and available at https://gitlab.com/mdoa-group/mpa-cloud-server/-/tree/master/src/main/java/service/omex/algorithmtest).

**Table 1:** Overview on the Performance of Filter, Wrapper, and the Combined Stages to Select Relevant Molecules

stage	precision	recall
filter only	0.75	1.0
wrapper only	0.88	0.63
filter + wrapper (OMEx)	0.91	0.76

For each synthetic data set, “relevant” molecules were known, providing a ground truth to evaluate the selection of molecules in the p-value filter step, wrapper step, and their combination. The selection of relevant molecules was evaluated (Table 1) by precision (a measure for the selection of true positive molecules) and recall (a measure for the “recovery” of true positive molecules). These metrics range from 0 to 1, with higher values indicating better performance (see the Supporting Information for a more thorough explanation).

The filter showed a high recall of 1.0, indicating that it reports all relevant molecules, while selecting many irrelevant ones, as indicated by the intermediate precision of 0.75. The wrapper can sort out irrelevant molecules and reliably report relevant ones, as indicated by its high precision of 0.88. However, it showed a low recall of 0.63, indicating that not all relevant features are found. The combination of both approaches had a recall of 0.76 and a precision of 0.91. Therefore, combining both stages shows a better recovery of relevant molecules and fewer false positive selections compared to the individual stages.

### OMEx Reproduces Selected Biomarkers from a Metaproteomics Data Set

Testing with synthetic data provided confidence in the algorithm on a theoretical level. To evaluate OMEx under realistic conditions, we collected and analyzed five data sets containing real omics data (Table 2).

**Table 2:** Overview on the Datasets Used to Test OMEx

data set	molecules (rows)	groups (#samples)	publication
stool metaproteomics	42,572	control (19), nonalcoholic steatohepatitis (32), hepatocellular carcinoma (29)	^8^
blood transcriptomics	10,527	control (35), rheumatoid arthritis (45)	^10^ (data set from Ng et al.^4^ was used)
blood proteomics	1070	control (35), rheumatoid arthritis (44)	^10^
urine metabolomics	2944	control (469), lung cancer (536)	^11^
glial tumor metabolomics	7017	IDH wild-type tumors (50), IDH mutant tumors (38)	^12^

The initial version of OMEx’s algorithm has been developed for the study by Sydor et al.,^8^ who determined potential biomarkers for nonalcoholic steatohepatitis (NASH) and hepatocellular carcinoma (HCC) from metaproteomics of stool samples. The current version (0.2.0) of OMEx was applied to this data set (stool metaproteomics data set) to test whether molecules determined for group pairings (control vs NASH, control vs HCC, NASH vs HCC) could be reproduced. Sydor et al. applied their algorithm to each group pairing individually, as well as on all three groups.^8^ Because OMEx currently supports analysis of only two groups, only results from group pairings were reproduced. Due to the random assignment of samples during steps 2 and 3, the most frequently selected molecules can differ slightly in every run. Therefore, the top 20 molecules from OMEx were compared to the reported biomarker candidates by Sydor et al.^8^ The most frequently reported molecule panel (a subset of the top 20 molecules) was used for a final classification run. Classification accuracy was determined by repeated cross-validation (1000 repeats). The classification accuracy describes the ratio of correctly classified samples compared with all classified samples. Accuracy ranges from 0 to 1, with a value of 1 meaning that all samples were classified correctly.

OMEx’s top 20 molecules reproduced many of the biomarkers from Sydor et al.,^8^ demonstrating that the top molecules are reported consistently (Table 3). The most frequently reported molecule panels resulted in high classification accuracies of the control and disease groups (0.99 and 0.99 accuracy for Control vs NASH and Control vs HCC, respectively) and a lower accuracy for the two disease groups (0.75 accuracy for NASH vs HCC), which is in line with the original study (Table 3). For this pairing, OMEx’s accuracy was lower than that in Sydor et al. (OMEx: 0.75, Sydor et al.: 0.86).^8^ This potentially originates from a different feature selection strategy, resulting in two molecules and seven molecules used in the classification by OMEx and Sydor et al., respectively.^8^

**Table 3:** Reproduction of Results From the Original Algorithm

	control vs NASH	control vs HCC	NASH vs HCC
reproduced biomarkers (OMEx/original)	6/7	5/5	7/10
size of selected panel by OMEx	4	3	2

	classification accuracies		
original algorithm (cross-validation)	0.99	1.00	0.86
OMEx (cross-validation)	0.99	0.99	0.75

### Comparison of OMEx to Other Molecule Selection Methods

Furthermore, four publicly available omics data sets of different sizes were collected (Table 2). These data sets had been used in several other studies applying different feature selection methods allowing for a benchmarking of OMEx’s outputs.

Tasaki et al.^10^ performed a multiomics study of rheumatoid arthritis (RA) applying transcriptomics and proteomics analyses to blood samples from patients (blood transcriptomics and blood proteomics data set). They created ensembles of partial least-squares regression (PLSR) models for each data set and reported the molecules most influential on model output. The blood transcriptomics data set was used by Ng et al.^4^ in a tutorial on biomarker discovery. They applied XGBoost, an algorithm based on decision trees, and extracted the most important molecules for their model using the Shapley Additive Explanations (SHAP).

The urine metabolomics data set contains metabolomics measurements from patients with lung cancer.^11^ The authors applied random forests to extract potential biomarkers, while other studies by Chardin et al.^12^ and Labory et al.^3^ performed biomarker selection using supervised autoencoders (SAE) and a combination of Boruta feature selection and partial least-squares discriminant analysis (PLS-LDA) respectively.

Chardin et al.^12^ also provided a metabolomics data set on glial tumors (glial tumor metabolomics data set) containing wild-type and isocitrate dehydrogenase (IDH) mutants used for the classification of glial tumors. SAE and Boruta combined with PLS-LDA were also applied for biomarker selection from this data set by Chardin et al.^12^ and Labory et al.,^3^ respectively.

All data sets, OMEx configurations, and results are available in the supplementary files. All data sets were benchmarked by applying the automated mode of OMEx. For the urine metabolomics data set, 70% of the samples were used for molecule selection, and 30% to evaluate the classification performance based on accuracy. For other data sets, classification performance was evaluated by repeated cross-validation (1000 repeats).

OMEx provides a ranking of molecules based on the frequency of selections in the wrapper stage. For the blood transcriptomics and blood proteomics data set, the top 20 molecules were compared to reported molecules from other studies, whereas only the top four and top five molecules were considered for the urine metabolomics and glial tumor metabolomics data sets, respectively, because Mathé et al.^11^ and Chardin et al.^12^ did not provide more molecules (Figure 3).

**Figure 3:**
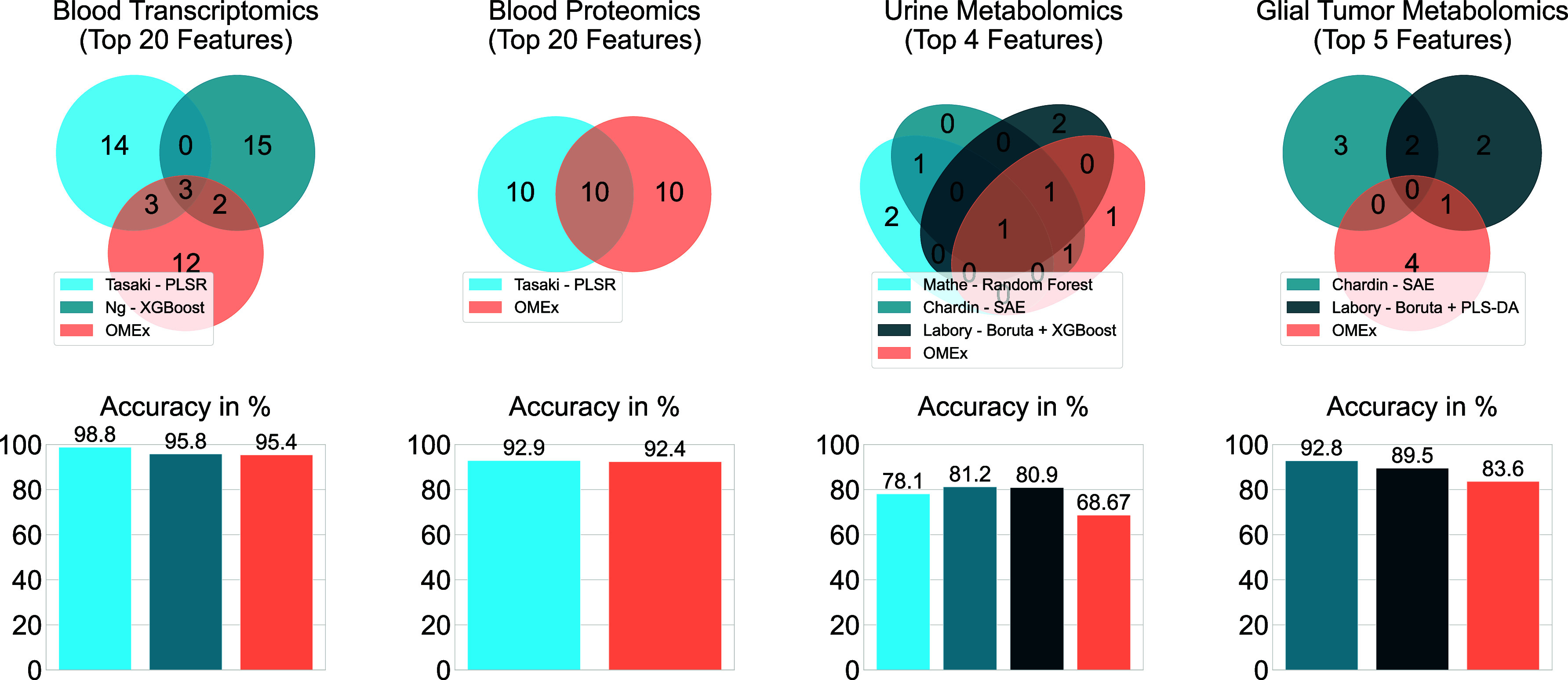
OMEx and other methods applied to four omics data sets. Results from other tools were obtained from the respective publications. Venn diagrams show overlaps between the *n* most important molecules as reported by each method (Chardin et al.^12^ reported only the top five molecules, Mathé et al.^11^ did only report top four molecules). Bar plots show the classification accuracy, assessed using cross-validation for all data sets except the urine metabolomics data set, which was evaluated using an independent test set.

OMEx’s top reported molecules overlapped with all other methods except for the SAE on the glial tumor metabolomics data set. Potentially, more reported molecules would have resulted in a higher overlap. These results show that OMEx can extract the most important molecules, also covering alternative molecules that are potentially not considered by other methods.

Aside from molecule rankings, OMEx provides molecule panels that contain a few molecules and are therefore suitable for experimental validation. The best panels for each data set contained between one and five molecules (Table 4). Selected transcripts or proteins were annotated by querying UniProt,^13^ and metabolites were annotated using the Workflow4Metabolomics platform^14^ (Supporting Information). A classification based on these panels was compared to the other methods using accuracy, as this was the metric reported by all other methods.

**Table 4:** Most Frequently Selected Molecule Panels by OMEx for the Four Benchmarking Datasets

data set	best panel with identifiers from data set	molecule names
blood transcriptomics	FAM160B1	FHF complex subunit HOOK interacting protein 2A (UniProt: Q5W0 V3)
	KHSRP	far upstream element-binding protein 2 (UniProt: Q92945)
blood proteomics	CKB CKM	creatine kinase M-type (UniProt: P06732), creatine kinase B-type (UniProt: P12277)
	CTSS	cathepsin S (UniProt: P25774)
urine metabolomics	MZ 238.00	4-chloro-5-(2-chloroethenyl)-1-(chloromethyl)-5-methylcyclohexene
	MZ 264.12	(*Z*)-2-methyl-2-butene-1,4-diol 4-*O*-beta-d-glucopyranoside
	MZ 208.06	3,5-dimethyl-2-(3-pyridyl)thiazolidin-4-one
	MZ 307.02	nitazoxanide
glial tumor metabolomics	MZ 173.03	*N*-formyl-l-glutamate

In all data sets, the classification accuracy across all methods was between 83.6% and 98.8%, indicating that most samples could be separated. On the urine data set, methods showed a drop in accuracy between 68.67 and 81.2%. Overall, OMEx performs comparably to other methods, but it did not outperform them. Additionally, we repeated these analyses using Random Forest and OPLS-DA for comparison with OMEx, yielding similar results (see Supporting Information, Section 7). Furthermore, we reproduced the molecule selection by Reinders et al.,^15^ demonstrating that the panels identified by OMEx can generalize to independent sample cohorts (see Supporting Information, Section 8). It must be noted that the main objective of OMEx is not classification, but molecule selection. Additionally, OMEx utilizes a very simple classification algorithm that is less prone to overfitting compared to the more advanced methods such as random forests, XGBoost, and SAE.

## Conclusions

Overall, OMEx provides an excellent solution for the selection of molecule panels as biomarker candidates from large matrices of omics data. The method, embedding diagonal linear discriminant analysis into the combination of filter and wrapper techniques, represents a useful strategy to extract biomarker candidates reliably, as shown on synthetic data. While outcomes of OMEx can be slightly different in each run, it was shown that biomarkers reported by a previous version of the algorithm were in accordance with OMEx, demonstrating the reproducibility of the tool results. Additionally, OMEx selects molecules similar to those reported by other computational approaches while also selecting unique molecules that others have not, demonstrating its value as an alternative approach. The most striking advantage of OMEx, except for its novel algorithm, is its user-friendly and web-based interface, minimizing the effort to analyze omics data and obtain potential biomarkers for experimental validation. In the future, OMEx will be extended to extract molecules for more than two groups and provide more advanced classification models.

## Supplementary Material





## Data Availability

All data sets used in this manuscript correspond to the output of raw data processing, i.e., list of molecules with estimated abundances. We retrieved these data sets from Supporting Information/repositories related to their original publications or the original authors (Table 2). These data sets were transferred into OMEx’s input format. The original raw data of each data set and processed data can be retrieved from Stool metaproteomics: PRIDE (https://www.ebi.ac.uk/pride/, ID PXD034175), processed data were provided by authors;^8^ Blood transcriptomics: GEO (Home - GEO - NCBI, ID GSE93777), processed data retrieved from https://github.com/C4TB/CTR_XAI, Blood proteomics: raw and processed data can be retrieved from Synapse (https://www.synapse.org/Home:x, ID syn8483403); Urine metabolomics: Metabolights (https://www.ebi.ac.uk/metabolights/, ID MTBLS28), procesed data retrieved from https://github.com/Plant-Net/Metabolomic_project/tree/d333a516e378ad5e8420eb9b1afbf3ccd81b9667; Glial tumor metabolomics: No raw data were made available by the authors; the data set used is available on GitHub (https://github.com/CyprienGille/Supervised-Autoencoder); and Lymphoma proteomics: ProteomeXchange (https://www.proteomexchange.org/, ID PXD012317), processed data were provided by the authors.^15^

## Notes

Datasets_formatted: All data sets used in the manuscript formatted to comply OMEx input format; omex_sydor_comparison: results generated by OMEx for the comparison to its original implementation by Sydor et al.^8^ and overview on overlapping molecules; benchmarking_results: all results generated by OMEx for the comparison with other molecule selection methods and R scripts and results for RF and OPLS-DA analyses; selected_molecule_identification: output from Workbench4Metabolomics^14^ and UniProt^13^ queries for the annotation of molecule panels found in the comparison of molecule selection methods; biomarker_validation: data sets in OMEx’s input format and results generated for the reproduction of the molecule selection workflow of Reinders et al.^15^ (ZIP)
Figure S1: Diagram of the proof-of-concept study of OMEx’s algorithm; Figure S2: algorithm description for generating synthetic data sets; Figure S3: distributions of molecules produced by the sampling algorithm for generating synthetic data sets; Figure S4: screenshot of OMEx’s user interface for the first workflow step; Figure S5: screenshot of the data table containing the stool metaproteomics data; Figure S6: data overview figures; Figure S7: filter results figure; Figure S8: wrapper results figure; Figure S9: molecule panel selection table; Figure S10: classification figures overview; Figure S11: performance comparison of all molecule selection/classification methods; Figures S12–S15: upset plots showing the intersections of selected molecule panels (PDF)
