# Supplementary figures and images for: The Omics Molecule Extractor: A Web Application for the Selection of Potential Biomarker Panels
Source: J Proteome Res. 2025 Dec 1;25(1):484–90. doi: 10.1021/acs.jproteome.5c00176 (PMC12772118; doi:10.1021/acs.jproteome.5c00176)

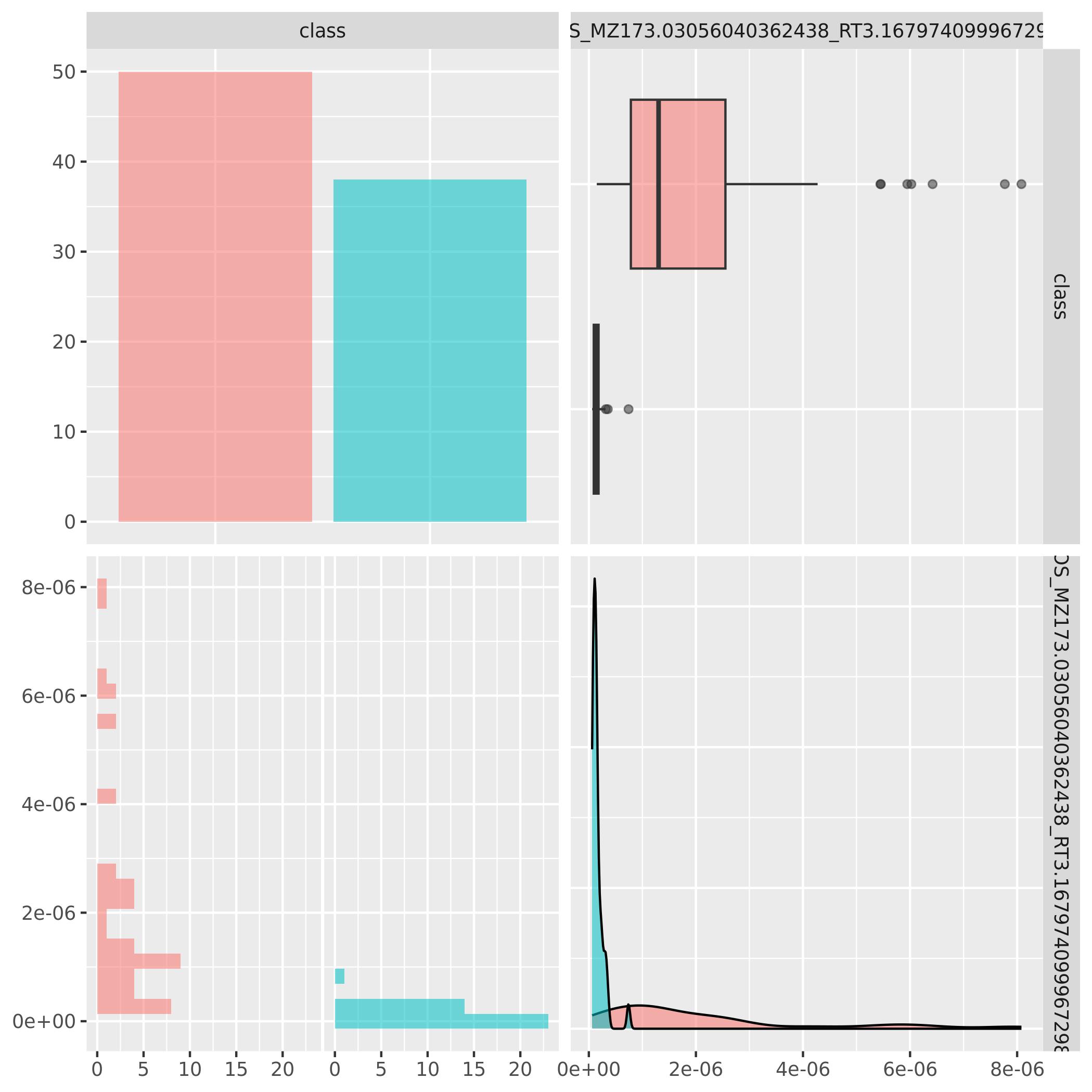

Supplement: Supplementary file 1 [file pr5c00176_si_001.zip › supplementary_data/benchmarking_results/chardin_brain_cv/scatterPlotImage.jpg]

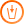

Supplement: Supplementary file 1 [file pr5c00176_si_001.zip › supplementary_data/benchmarking_results/mathe_lung_with_testset/hacImage_files/vis-9.1.0/img/network/acceptDeleteIcon.png]

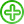

Supplement: Supplementary file 1 [file pr5c00176_si_001.zip › supplementary_data/benchmarking_results/mathe_lung_with_testset/hacImage_files/vis-9.1.0/img/network/addNodeIcon.png]

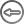

Supplement: Supplementary file 1 [file pr5c00176_si_001.zip › supplementary_data/benchmarking_results/mathe_lung_with_testset/hacImage_files/vis-9.1.0/img/network/backIcon.png]

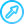

Supplement: Supplementary file 1 [file pr5c00176_si_001.zip › supplementary_data/benchmarking_results/mathe_lung_with_testset/hacImage_files/vis-9.1.0/img/network/connectIcon.png]

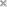

Supplement: Supplementary file 1 [file pr5c00176_si_001.zip › supplementary_data/benchmarking_results/mathe_lung_with_testset/hacImage_files/vis-9.1.0/img/network/cross.png]

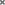

Supplement: Supplementary file 1 [file pr5c00176_si_001.zip › supplementary_data/benchmarking_results/mathe_lung_with_testset/hacImage_files/vis-9.1.0/img/network/cross2.png]

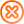

Supplement: Supplementary file 1 [file pr5c00176_si_001.zip › supplementary_data/benchmarking_results/mathe_lung_with_testset/hacImage_files/vis-9.1.0/img/network/deleteIcon.png]

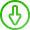

Supplement: Supplementary file 1 [file pr5c00176_si_001.zip › supplementary_data/benchmarking_results/mathe_lung_with_testset/hacImage_files/vis-9.1.0/img/network/downArrow.png]

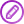

Supplement: Supplementary file 1 [file pr5c00176_si_001.zip › supplementary_data/benchmarking_results/mathe_lung_with_testset/hacImage_files/vis-9.1.0/img/network/editIcon.png]

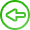

Supplement: Supplementary file 1 [file pr5c00176_si_001.zip › supplementary_data/benchmarking_results/mathe_lung_with_testset/hacImage_files/vis-9.1.0/img/network/leftArrow.png]

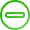

Supplement: Supplementary file 1 [file pr5c00176_si_001.zip › supplementary_data/benchmarking_results/mathe_lung_with_testset/hacImage_files/vis-9.1.0/img/network/minus.png]

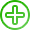

Supplement: Supplementary file 1 [file pr5c00176_si_001.zip › supplementary_data/benchmarking_results/mathe_lung_with_testset/hacImage_files/vis-9.1.0/img/network/plus.png]

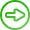

Supplement: Supplementary file 1 [file pr5c00176_si_001.zip › supplementary_data/benchmarking_results/mathe_lung_with_testset/hacImage_files/vis-9.1.0/img/network/rightArrow.png]

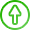

Supplement: Supplementary file 1 [file pr5c00176_si_001.zip › supplementary_data/benchmarking_results/mathe_lung_with_testset/hacImage_files/vis-9.1.0/img/network/upArrow.png]

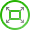

Supplement: Supplementary file 1 [file pr5c00176_si_001.zip › supplementary_data/benchmarking_results/mathe_lung_with_testset/hacImage_files/vis-9.1.0/img/network/zoomExtends.png]
