# Supplementary material for: The Omics Molecule Extractor: A Web Application for the Selection of Potential Biomarker Panels
Source: J Proteome Res. 2025 Dec 1;25(1):484–90. doi: 10.1021/acs.jproteome.5c00176 (PMC12772118; doi:10.1021/acs.jproteome.5c00176)
Supplement: Supplementary file 2 [file pr5c00176_si_002.pdf]

Supporting Information

The Omics Molecule Extractor: A web application for the selection of potential biomarker panels

Emanuel Lange[1,2,‡,*], Kay Schallert[1,‡], Johannes Schwerdt[3], Susmita Ghosh[1], Andreas Hentschel[1], Yvonne Reinders[1], Robert Heyer[1,4]

1 Leibniz-Institut für Analytische Wissenschaften - ISAS - e.V., 44139 Dortmund, Germany

2 Graduate School Digital Infrastructure for the Life Sciences, Bielefeld University, 33615 Bielefeld, Germany

3 Hochschule Merseburg, 06217 Merseburg, Germany

4 Multidimensional Omics Data Analysis, Faculty of Technology, Bielefeld University, 33615 Bielefeld, Germany

Corresponding author email: emanuel.lange@isas.de

1. List of supplementary Files

- supplementary.docx: Supplementary information to main manuscript
- supplementary_data.zip: all supplementary data for the manuscript including following subdirectories:
    - datasets_formatted: All datasets used in the manuscript formatted to comply OMEx input format
    - omex_sydor_comparison: Results generated for the comparison of OMEx and its original implementation by Sydor *et al.*[1]
    - benchmarking_results: All results generated by OMEx for the comparison with other molecule selection methods
    - selected_molecule_identification: Output from Workbench4Metabolomics[2] and UniProt[3] queries for the annotation of molecule panels found in the comparison of molecule selection methods
    - biomarker_validation: All results generated by OMEx for the reproduction of the molecule selection workflow by Reinders *et al.*[4]

2. Detailed description of OMEx Algorithm

The Omics Molecule Extractor combines different machine-learning techniques (figure 1). The main objective is to determine a small set of molecules allowing for the best separation of samples (e.g., patients) into their associated categories, for example, control and disease. Diagonal Linear Discriminant Analysis (d-LDA) is used for classification, which has proven to be computationally lightweight but sufficiently accurate on a metaproteomics dataset compared to other classification methods [1]. Molecules for classification are selected based on a p-value filter and a wrapper[1].

The p-value filter performs a statistical test (two-sample t-test) on each molecule independently, to determine molecules with significantly different magnitudes among the categories. The greater the difference in the molecule distribution between the classes, the smaller the p-value of the test. Subsequently, features below a specified p-value cutoff are filtered and provided to the wrapper. The selection of molecules is done in a five-fold cross-validation setting, i.e., four folds of randomly selected samples are used to determine p-values and train the classifier, and one fold is used to test the classifier accuracy. This process is done five times, iterating through the five folds so that each fold has been used for testing once, while remaining folds were used for training, respectively. Classification accuracy is evaluated at different p-value cutoffs (e.g., 0.0001, 0.001, 0.005, etc.) to assess an appropriate cutoff value. Lastly, the average number of selected molecules and average accuracy are reported for each cutoff.

The wrapper iteratively adds molecules to a panel of molecules used for classification. Firstly, the input dataset is divided randomly into five cross-validation folds, of which four are used for training and one for testing. Using data from the training fold, the wrapper starts with molecules with low p-values, adds them to a panel, trains the classifier on this panel, determines classification accuracy using the test fold, repeats until the classifier accuracy does not increase anymore, and reports how often specific molecule panels and individual molecules were chosen by the classifier. Usually, this results in panels containing less than ten molecules. Subsequently, the training and test sets shift to the next fold, and the process is repeated until the complete dataset has been used for testing. As samples are assigned randomly to their folds, the output of each wrapper run can be different, but well-separating molecules will be chosen more likely than others. By repeating the cross-validation step, for example 1,000 times, panels and individual molecules can be ranked by how often they were picked by the classifier.

An advantage of the wrapper over p-value filtering is that combinations of molecules are considered in the classification. However, wrapping is a computationally expensive technique. Therefore, the preceding p-value filtering reduces the total computation time. The most frequently chosen panels/molecules are considered to be potential biomarkers. As a last step, independent test samples are classified using the selected molecules to evaluate their predictive power based on classification metrics (accuracy, precision, recall, f1 score). Additionally, a Principal Component Analysis (PCA) and hierarchical clustering are performed based on the selected molecules to visualize their capability of separating the groups.

3. Creation of synthetic Datasets

To validate the applicability of the proposed tool, we applied synthetic data to measure the detection rate of relevant molecules identified by our tool. Therefore, we created a synthetic data-set generator that creates several measurement matrices that consist of two groups (e.g., healthy and disease) encoded in the columns and several molecules representing molecules encoded in the rows. Values in some rows will be systematically differently distributed between groupings, the relevant molecules, and values in other rows will be equally distributed between groupings, the non-relevant molecules. The measurement matrix will be presented to the OMEx modules to identify the (hidden) relevant molecule measurements. Subsequently, the prediction will be compared to the ground-truth information from the synthetic data-set generator to evaluate the detection rate. It has to be stated in all clarity that real-life processes are more complex. This artificial evaluation rather indicates the usefulness of the tool in a theoretical/hypothetical sense. Therefore, additional verification of the performance on real-life data is needed, and it can be found in the main paper. The described approach is depicted in figure S1.

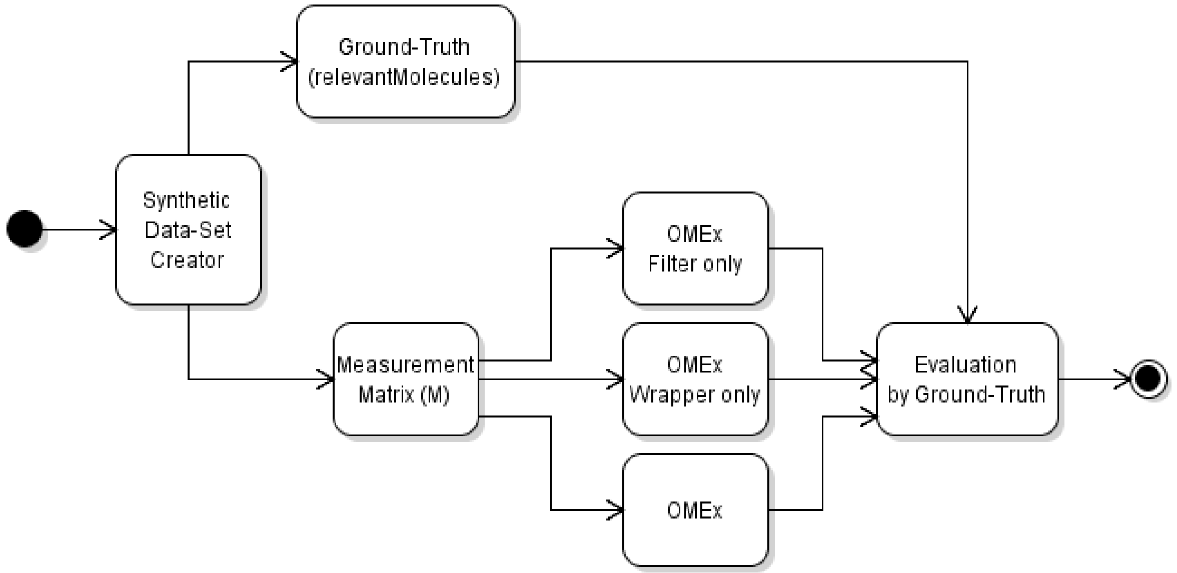

Figure S1: The diagram of the proof-of-concept study of the proposed OMEx tool using a synthetic data set generator.

The artificial data generator (see pseudocode in figure S2) is based on exploiting sequences of (pseudo) random numbers. This technique is also known as sampling. For the sampling sequence, we need to introduce some restricting assumptions. Each data point belongs to exactly one group/class, and this assignment follows a Bernoulli-Distribution [B], in case of two classes, e.g., C1 and C2. The balance of these groupings is encoded by the distribution's parameter that is connected with its Conjugate Prior (a Dirichlet-Distribution [Dir]). Each molecule within the data set belongs to a latent grouping, being either relevant or non-relevant. Therefore, this assignment follows another Bernoulli-

Distribution [B] with a distribution's parameter conjugated to its own Conjugate Prior (a Dirichlet-Distribution [Dir]). We further assume that molecules are drawn from Normal-Distributions [N]. In the case of non-relevant molecules, they follow a shared distribution N(μ_0, σ_0); In the case of relevant molecules, they follow class-specific distributions N(μ_c1, σ_c1) and N(μ_c2, σ_c2) (figure S3). The distribution parameters for μ's and σ's can be adjusted by the user. We choose the simple parametrization that can be found in figure S2. Further, we restrict sampled values to be non-negative by setting negative samples to zero. This step was incorporated to emulate missing measurements indicated by the zero, exactly as it can be found in the real-life data sets used in our work. With these basic assumptions set, we can create sampled datasets to evaluate the proposed tool by exploiting the information stored about the sampling process. By repeating this procedure multiple times, one can create several artificially generated data sets to evaluate the molecule selection algorithm.

```
procedure DATA SET SAMPLER

    Initialisation:
        set dataSetSize                                              (e.g. to 60)
        set moleculeSize                                             (e.g. to 50)
        set Relevance-Prior α_rel = (α_R, α_NR)^T                    (e.g. 10,1000)
        set Class-Balance-Prior α_class = (α_c1, α_c2)^T             (e.g. 50,50)
        set Measurement-Priors μ_c1, μ_c2, μ_0, σ_c1, σ_c2, σ_0      (e.g. 2,0,1,1,1,1)

    Generator:
        relevanceProportion ~ Dir(α_rel)
        classProportion ~ Dir(α_class)

        relevantMolecules = List()
        C_1 = Matrix(moleculeSize, ⌊classProportion_1 · dataSetSize⌋)
        C_2 = Matrix(moleculeSize, ⌊classProportion_2 · dataSetSize⌋)
        M = C_1 ⊗ C_2 = Matrix(moleculeSize, dataSetSize)

        foreach(molecule ∈ moleculeSize)

            isRelevant ~ B(relevanceProportion)
            if(isRelevant)
                relevantMolecules.append(molecule)

            foreach(measurement ∈ dataSetSize)
                if(isRelevant)
                    if(measurement ∈ C_1)
                        M(molecule, measurement) ~ max(0, N(μ_c1, σ_c1))
                    else
                        M(molecule, measurement) ~ max(0, N(μ_c2, σ_c2))
                else
                    M(molecule, measurement) ~ max(0, N(μ_0, σ_0))

        return(M, relevantMolecules)
```

Figure S2 The function generates a sampled data set for the described properties. A tupel will be returned for the measurement matrix itself and a list of the relevant molecules.

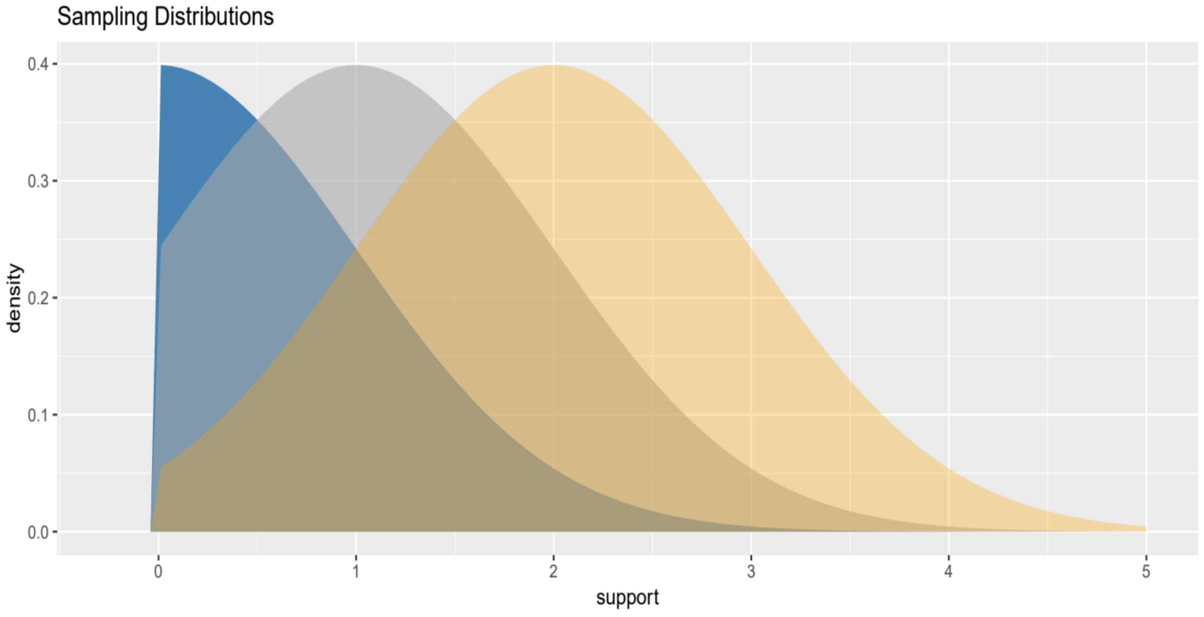

Figure S3 Overlapping distribution of molecules produced by the sampler. The x-axis (support) corresponds to the values that can be sampled for artificial molecules, the y-axis (density) corresponds to the probability of sampling one specific value. Non-relevant molecules share the gray distribution in the middle across both groups. Relevant molecules have class-specific distributions (blue & yellow). All distributions share a reasonable overlap, making the task not unrealistically easy.

4. Evaluation of the algorithm using synthetic datasets

We applied the sampling approach from section 2 to generate artificial data sets for an initial evaluation of the OMEx modules individually and in combination (https://gitlab.com/kay.schallert/mpa-cloud-server/-/tree/master/src/main/java/service/omex/algorithmtest). All results presented are produced by the parametrization depicted in figure S2 and figure S3 exemplary. Within the analysis, 100 artificial data sets were generated. They were restricted to exactly 60 samples (artificial participants, encoded by the variable dataSetSize) with 50 artificial molecules (encoded by moleculeSize). We enforced an equal class balance by setting $\alpha\_class = (50, 50)$. In addition, the balance of the molecule relevance is strongly enforced to be unequal in favor of non-relevant molecules by setting $\alpha\_rel = (10, 100)$. The molecule generating distribution was set to three distinct Normal Distributions, with a shared $\mu\_0 = 1$ for the non-relevant molecules and $\mu\_c1 = 0$ & $\mu\_c2 = 2$ for the class-specific and relevant molecules, respectively. All three distributions are instantiated with the unit variance. This results in distinct yet strongly overlapping boundaries (figure S3).

The generated synthetic datasets were subjected to the filter and wrapper individually, as well as to their combination. The numbers of molecules predicted as “relevant” (positive) and “non-relevant” (negative) by each stage were compared to their ground-truth groupings resulting in a confusion matrix (table S1).

Table S1 Structure of a confusion matrix.

		prediction	
		relevant	non-relevant
ground-truth	relevant	# true positive predictions (TP)	# false negative predictions (FN)
	non-relevant	# false positive predictions (FP)	# true negative predictions (TN)

From this comparison, we calculated the precision, and recall. Precision (equation 1) describes whether members predicted as positives (i.e., relevant molecules) are correct (equation 2), and recall describes the capability to find all members of the positive group (equation 3).

$$Precision = \frac{true\ positive\ predictions}{all\ positive\ predictions} = \frac{TP}{TP+FP} \qquad (1)$$

$$Recall = \frac{true\ positive\ predictions}{ground\ truth\ positives} = \frac{TP}{TP+FN} \qquad (2)$$

All three metrics describe ratios and range from 0 to 1 (the higher, the better). A precision of 0 for example, means that only non-relevant molecules were predicted as relevant, while 1 means that all predicted relevant molecules are indeed relevant. Likewise, a recall of 1 means that all relevant molecules were predicted as relevant, 0 means all relevant molecules were predicted as non-relevant.

4.1 OMEx – Filter only

The derived confusion matrix for the filter-only strategy can be found in table S2. The overall accuracy of the filter is high (0.97), however it rather reports too many features as too few, as demonstrated by the high recall of 1 and the low precision of 0.75. These extreme results originate from the near optimal design of the synthetic data set generator (only white-noise, no additional 'hidden' processes, 'statistically perfect' distributions, etc.). Yet, these results also imply that automatic thresholding by classification accuracy is a viable selection strategy and that the implementation of the proposed tool works in its intended way.

Table S2 Confusion matrix derived from the sampling and the filter stage. Entries are the cumulated results of molecule selections from 100 datasets including 50 molecules and 60 samples. The filter stage rather reports too many molecules as too few.

		prediction	
		relevant	non-relevant
ground-truth	relevant	440	0
	non-relevant	141	4,419

4.2 OMEx – Wrapper only

The derived confusion matrix for the wrapper-only strategy can be found in table S3. Still the accuracy is high with a value of 0.96 but it can be stated that the wrapper rather reports too few features as too many, as demonstrated by the low recall of 0.63 and high precision of 0.88. These results indicate that this molecule selection is a suboptimal strategy to identify all relevant features. Even though, the precision is considerably higher compared to the filter, most of the relevant features will not be reported. Furthermore, the computing time is significantly higher and becomes not practical for more than 150 molecules.

Table S3 Confusion matrix derived from the sampling and the wrapper approach. Entries are the cumulated results of molecule selections from 100 datasets including 50 molecules and 60 samples. The wrapper stage rather reports too few molecules as too many.

		prediction	
		relevant	non-relevant
ground-truth	relevant	280	160
	non-relevant	35	4,525

4.3 OMEx (combined Filter & Wrapper)

The derived Confusion Matrix for OMEx (combined strategy of filter and wrapper) can be found in table S4. The results of section 3.1 and 3.2 implied that the filter and the wrapper strategies comprise

complementary strengths and weaknesses. Therefore, an initial filter was applied in a (pre-)filtering stage. The p-value threshold value for filtering was selected by maximal accuracy. The remaining molecules were provided to the wrapper stage for a (post-)filtering. The results indicate that the combined approach produces balanced results with a recall of 0.76 and precision of 0.91 at high accuracy of 0.97. Yet, the combined strategy tends to report rather too few molecules as too many. Therefore, users should inspect multiple high-ranking panels instead of exclusively concentrating on the top ranking one.

Table S4 Confusion matrix derived from the sampling and the combined approach of filter and wrapper (OMEx). Entries are the cumulated results of molecule selections from 100 datasets including 50 molecules and 60 samples. The combined approach produces balanced results compared to the individual strategies.

		prediction	
		relevant	non-relevant
ground-truth	relevant	338	102
	non-relevant	31	4,529

4 Benchmarking OMEx using five omics datasets

OMEx was tested and compared against its original implementation using a stool metaproteomics dataset[1] and against other molecule selection methods using blood proteomics[5], blood transcriptomics[5], urine metabolomics[6], and glial tumor metabolomics[7] datasets (see main manuscript for a detailed description of datasets). A processed version of the blood transcriptomics dataset was used, which had been used in another classification study[8]. All input datasets were formatted to comply OMEx input format (datasets_formatted.zip).

For every run with OMEx, the automatic mode was run based on the default parameters (table S5).

Table S5 Default parameters for every step of OMEx. [repeated analysis]

Parameter	value
Data input	
Non-sparsity fraction	0.66
Normalize data	true
split a test set from input data	false
Test set fraction	0.3
Filter	
folds	5
repeats	1000
Wrapper	
Molecule cutoff	As specified by OMEx
folds	5
repeats	1000

For the comparison to the original algorithm three group pairings were run (C vs. NASH, C vs. HCC, NASH vs. HCC) with default parameters and using cross validation for performance evaluation.

For the blood proteomics, urine metabolomics and glial tumor metabolomics datasets, default parameters were used. For the blood transcriptomics dataset normalization was disabled. For the lung metabolomics dataset, a training/testing split of 70%/30% was used.

Configurations can also be inspected when opening the config.json file contained in the output directory of every OMEx run (omex_sydor_comparison.zip, benchmarking_results.zip, biomarker_validation.zip).

5 Annotation of molecule panels

We provided the selected panels for the blood proteomics, blood transcriptomics, urine metabolomics and glial tumor metabolomics datasets in the main manuscript. No meta information on molecules was contained in the datasets requiring a manual annotation of molecule identifiers.

For the blood proteomics and blood transcriptomics datasets, gene names were contained in the datasets, which were queried in UniProt[3] (date of access: August 6, 2025). For the two metabolomics datasets, molecule m/z values were present in molecule identifiers and could be input into the Annotation LCMS FIAMS tool on the Workflow4Metabolomics platform[2]. Specifically, the “bank-inhouse” tool was used to annotate m/z values. Parameters can be found in the subdirectories for the respective datasets in “selected_molecule_identification.zip”.

6 Example Case Study: Stool Metaproteomics

This case study should provide an overview on how to interpret the parameters and outputs of OMEx. We use the Stool Metaproteomics dataset as an example. This example can be reproduced by starting a new workflow, clicking on “select example data”, and selecting the “STOOL_METAPROTEOMICS” dataset.

User Interface

The main workflow of OMEx is represented as a vertical scrollable stepper (figure S4, A). With progressing in the workflow, by submitting parameters or retrieving results, the stepper successively extends towards the bottom of the page. Users are informed on the current status of the workflow through the status text and the progress bar at the bottom of the view (figure S4, B).

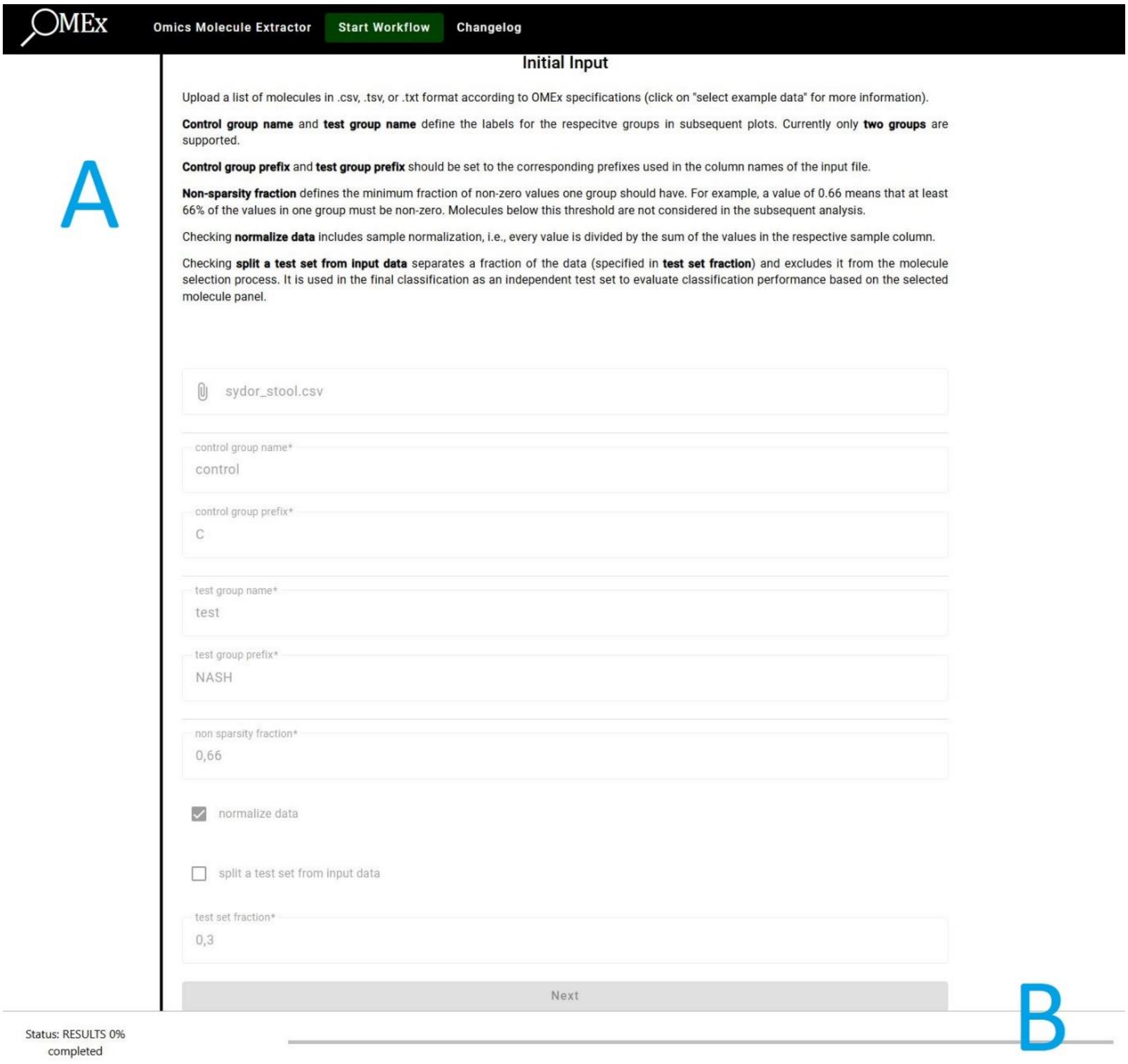

Figure S4: Screenshot of OMEx's user interface for the first workflow step. The workflow consists of a vertical, scrollable stepper that successively extends upon finishing the individual steps of the workflow (A). The status of the workflow is shown at the bottom through a status text and a progress bar (B).

Step 1) Initial input

The input format for the data table is a tab-separated .csv, .tsv, or .txt file containing molecule names in rows, sample names in columns and measured quantities in cells (figure S5). Sample column names are prefixed with condition names ("C_", "NASH_", and "HCC_" for control, non-alcoholic steatohepatitis, and hepatocellular carcinoma, respectively) for grouping. The very first column should include the descriptor, i.e., a unique identifier for each molecule (column name can be arbitrary). After the descriptor column any number of additional columns containing meta-information can follow. The sample columns should only include numeric data. If your input file does not comply OMEx's input format, a notification will be displayed, and data need to be re-uploaded.

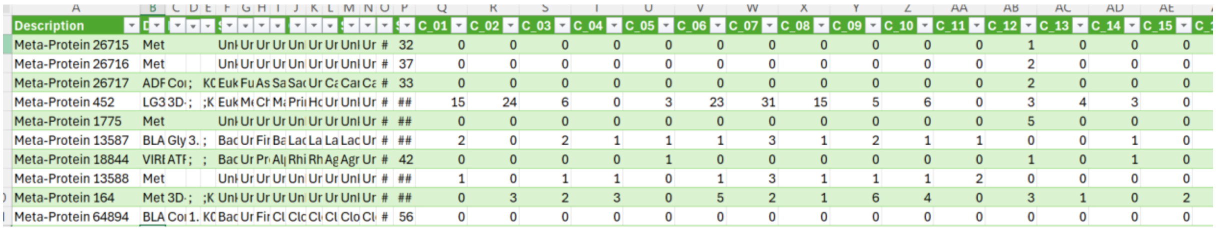

Description	D															C_01	C_02	C_03	C_04	C_05	C_06	C_07	C_08	C_09	C_10	C_11	C_12	C_13	C_14	C_15
Meta-Protein 26715	Met				Unk	Ur	Ur	Ur	Un	Ur	Ur	Unl	Ur	#	32	0	0	0	0	0	0	0	0	0	0	0	1	0	0	0
Meta-Protein 26716	Met				Unk	Ur	Ur	Ur	Un	Ur	Ur	Unl	Ur	#	37	0	0	0	0	0	0	0	0	0	0	0	2	0	0	0
Meta-Protein 26717	ADF	Coı	;	KC	Euk	Fu	As	Sa	Sac	Ur	Cá	Car	Cá	#	33	0	0	0	0	0	0	0	0	0	0	0	2	0	0	0
Meta-Protein 452	LG3	3D-	;	;K	Euk	Me	Ch	Mà	Prii	Hc	Ur	Unl	Ur	#	##	15	24	6	0	3	23	31	15	5	6	0	3	4	3	0
Meta-Protein 1775	Met				Unk	Ur	Ur	Ur	Un	Ur	Ur	Unl	Ur	#	##	0	0	0	0	0	0	0	0	0	0	0	5	0	0	0
Meta-Protein 13587	BLA	Gly	3.	;	Bac	Ur	Fir	Ba	Lac	La	La	Lac	Ur	#	##	2	0	2	1	1	1	3	1	2	1	1	0	0	1	0
Meta-Protein 18844	VIRI	ATF	;	;	Bac	Ur	Pr	Al	Rhi	Rh	Ag	Agr	Ur	#	42	0	0	0	0	1	0	0	0	0	0	0	1	0	1	0
Meta-Protein 13588	Met				Unk	Ur	Ur	Ur	Un	Ur	Ur	Unl	Ur	#	##	1	0	1	1	0	1	3	1	1	1	2	0	0	0	0
Meta-Protein 164	Met	3D-	;	;K	Unk	Ur	Ur	Ur	Un	Ur	Ur	Unl	Ur	#	##	0	3	2	3	0	5	2	1	6	4	0	3	1	0	2
Meta-Protein 64894	BLA	Coı	1.	KC	Bac	Ur	Fir	Cl	Clc	Cl	Cl	Clo	Cl	#	56	0	0	0	0	0	0	0	0	0	0	0	0	0	0	0

Figure S5: screenshot of the data table containing the stool metaproteomics data.

Upon inputting the data, the parameters are set:

- **Control Group Name:** "control" - This is the label that will appear in plots and results for the baseline group. This can be an arbitrary name.
- **Control Group Prefix:** "C" - Sample columns in the metaproteomics dataset that begin with this prefix will be treated as part of the control group.
- **Test Group Name:** "test" - This label will be used to represent the comparison group.
- **Test Group Prefix:** "NASH" - Columns beginning with the "NASH_" prefix will be assigned to the test group. OMEx will then compare the control group against the "NASH" group.
- **Non-sparsity fraction:** "0.66" - This sets a minimum threshold for data completeness. Molecules must have non-zero values in at least 66% of samples within at least one of the two groups to be included in the analysis. This removes molecules that are sparse, i.e., that appear only for few samples. The default value of 0.66 has proven as a good initial value for this parameter.
- **Normalize data:** "enabled" - When enabled, the values in each sample column are normalized by dividing each value by the column sum, so samples are comparable in scale. This dataset is not normalized; therefore, normalization is performed. For datasets that have already been normalized, this value should be disabled.
- **Split a test set from input data:** "disabled" – When enabled, samples for an independent test set are removed from the training set used for molecule selection. The test set is used to evaluate the molecule panel performance in the final classification run. Splitting off a test set is only recommended for large datasets (>1000 samples), because otherwise selected features can have large variation between repeated runs. For this dataset, it is therefore omitted, and classification performance will be evaluated by repeated cross validation.
- **Test set fraction:** "0.3" – Sets the size of the test set relative to the total dataset. Not used in this example.

After clicking on "Next", OMEx generates figures providing an overview of the data (figure S6).

- **A) Class Distribution:** Compares the number of samples in each group. In this case there is an imbalance towards the NASH group, as more samples are in this group.
- **B) Volcano Plot (Differential Expression):** Molecules far left or right (with large log2 fold-change > 2 and < 0.5) and high on the plot (high significance, -log10(p-value) > 3) are highlighted as being differentially expressed. There are several molecules both upregulated and downregulated in NASH compared to the control group, suggesting clear biological differences between groups. Only non-sparse molecules are considered (see next bullet point).
- **C) Data Sparsity Plot (Zero Values):** A cumulative distribution of molecules based on their presence (non-zero values) across all samples. x-axis: Fraction of samples in which a molecule is detected (non-zero). y-axis: Number of molecules meeting or exceeding that fraction. For this dataset many molecules are sparse: a large number have non-zero values in less than 25% of all samples. This validates the need for filtering based on the non-sparsity fraction (set

to 0.66 earlier), which helps reduce noise by excluding rarely observed molecules. In this case, all molecules below 0.66 are not considered for further analyses.

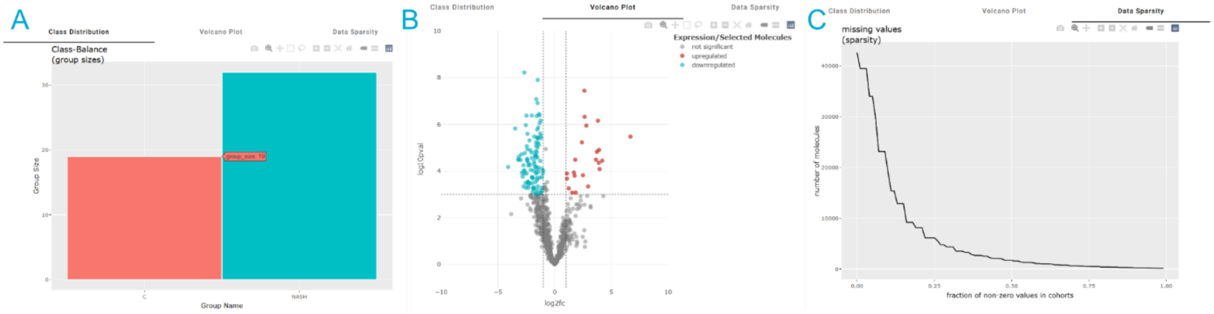

Figure S6 Data Overview figures. A – class sizes, B – Volcano Plot, C – Distribution of molecule sparsity.

Step 2) Filter

After obtaining an overview on the data, the first workflow step is performed, which determines the optimal number of molecules to be used in the wrapper step (step 3). Step 2 uses repeated cross-validation and p-value threshold tuning to later rank and select molecules. The objective is to reduce the number of molecules to an “important” subset, as the wrapper cannot handle many molecules.

- **Control Group**: “control”, Test Group “test” - These parameters should match the group names defined in the previous step.
- **Repeats**: “1000” - This means the entire cross-validation process will be repeated 1,000 times, each with a new random split of the data. This increases computation time but gives statistically robust results. Increasing this value is usually not necessary. It can be decreased to, e.g., 100, if the dataset is large (> 1000 samples) and has balanced group sizes to reduce computation time.
- **Folds**: “5” - The dataset will be split into 5 folds in each repetition: 4 folds used for classifier training and molecule selection, 1 fold used for testing the d-LDA classifier. This is repeated so each fold serves as a test set once per repetition. If datasets are large (>1000 samples) this parameter can be increased, e.g., to 10, which might improve generalization of the molecule panel and classifier.

After clicking on “Next”, OMEx evaluates different p-value cutoffs, resulting in different numbers of molecules for the next step (for example, 0.05 cutoff will provide 500 molecules and a cutoff of 0.001 will provide 100 molecules, figure S7). OMEx automatically selects the first cutoff producing less than 150 molecules.

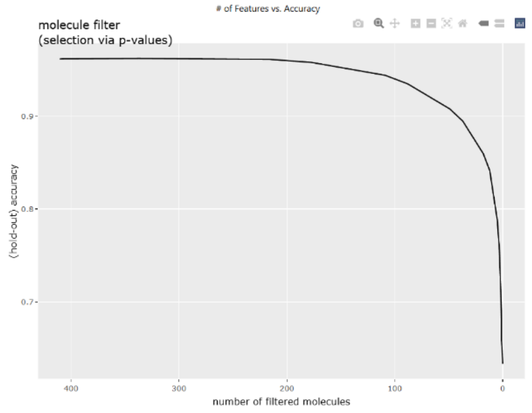

Figure S7 Classifier performance over different numbers of molecules determined through the p-value cutoff.

- **Classifier performance over number of features:** Figure S7 visualizes how classifier accuracy changes as features are added or removed by p-value thresholding. Inspect this graph if you want to determine the optimal number of molecules for the next step manually. This selection curve indicates that a small subset of molecules (~100–150) is sufficient to achieve near-maximum classification accuracy. Including more molecules does not yield meaningful gains.

Step 3) Wrapper

After filtering the initial data to a subset of < 150 molecules, these molecules are subjected to the wrapper. The wrapper determines potential molecule panels allowing to separate the samples into their groups and generates a ranking of these panels.

- **Molecule Cutoff:** „109“ – This is the number of top molecules (based on p-value ranking) that is subjected to the wrapper. OMEx automatically set the value to 109, based on the Filter step from before.
- **Repeats, Folds:** Cross validation repetitions and folds. See the same parameter description in the Filter step.

After clicking on “Next”, OMEx will generate multiple molecule panels (figure S8). This is the most time-consuming step and may take up to several hours for large datasets (e.g., the urine metabolomics dataset).

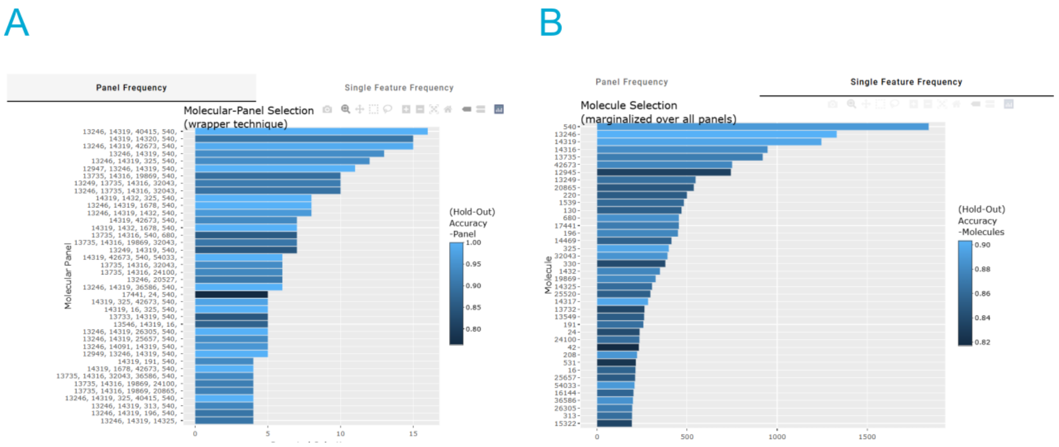

Figure S8 A -Molecule panel ranking and B – individual molecule ranking produced by the wrapper.

- **A) Panel Frequency:** Frequency (i.e., number of selections) and performance of entire molecule panels (combinations of molecules) selected during wrapper iterations. They y-axis lists the selected panels. In this case, the panel consisting of molecule 13246, 14319, 40415 and 540 has been selected most often in 1000 repetitions times 5 cross validation folds. The high frequency of selections means that it had the best performance across many random combinations of the training and test set during cross validation. Therefore, the most frequently selected panel is likely to generalize well. The bar color indicates the mean accuracy over all repetitions. If the best panel has a low average accuracy, it might make sense to select a different panel with satisfactory accuracy (however, this might not generalize as well as the most frequent panel).
- **B) Single Molecule Frequency:** Selection frequency of individual molecules summed across all panels that include this molecule. This ranking can be interpreted as “molecule importance”. Bar color represents the average accuracy of panels that include the individual

molecule. In this case, the three highest ranked individual molecules are also represented in the best panel.

Step 4) Panel Selection

This interface (figure S9) lets the user review molecules that were most frequently selected across wrapper repetitions, choose a subset of them to include in the final classification (profile selection drop down) and submit the selection for final performance evaluation. This step also allows to combine panels, e.g., by selecting the best and second-best panels from the drop-down menu. For the stool dataset, only molecules from the most frequently selected panel were chosen.

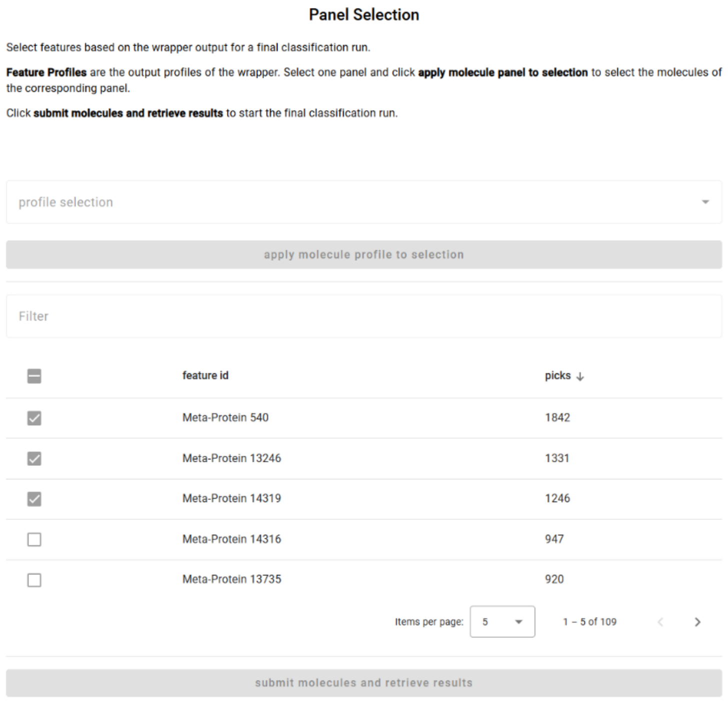

Figure S9: Selection of molecule panels that should be evaluated in the next step.

Step 5) Classification

The last step reports the evaluation of the selected molecule panel. It includes several visualizations to assess group separation, differences of individual molecules, and performance metrics of the d-LDA classifier trained on the molecule panel (figure S10). The classifier is evaluated on an independent test set if this has been enabled in step 1 or on the test sets in a cross validation setup otherwise.

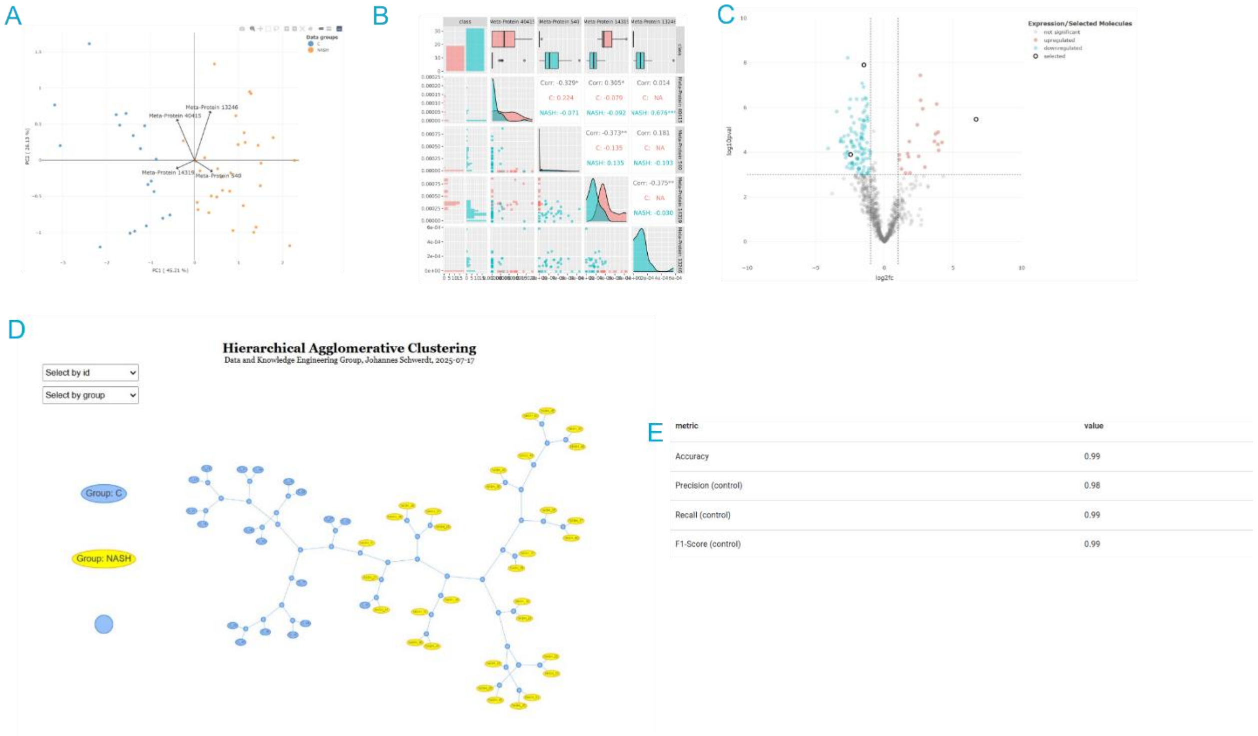

Figure S10: Output of the final classification. A – PCA plot, B – pairwise scatter plot, C – volcano plot, D – hierarchical clustering of samples, E – classifier performance metrics.

- **A) PCA Biplot (Principal Component Analysis):** Visualizes the separation between the two groups (C and NASH) based on the selected features. Samples are projected into principal component space. Vectors (arrows) represent top selected metaproteins (e.g., 540, 13246, etc.). There's clear separation between control and NASH groups, confirming that the selected molecules capture group-specific variation. The vectors also show the direction and contribution of each protein to the variance. Metaprotein 540 and Metaprotein 14319, for example, mostly explain variation across principle component 1 (x-axis).
- **B) Correlation and Distribution Matrix:** Displays pairwise correlations, group-wise distributions, and molecule-molecule relationships for the selected molecules. Diagonal: Histograms/density plots for each meta-protein. lower triangle: Scatter plots of pairwise relationships. Upper triangle: Correlation coefficients per group (C and NASH). This plot shows how features interact and differ across groups, and whether they carry redundant or complementary information. For example, Meta-Protein 14319 has a moderate positive correlation with 540 in NASH but not in controls.
- **C) Annotated Volcano Plot:** Revisits the volcano plot, now highlighting which molecules were selected for classification. Selected molecules are circled, clearly showing that they lie in statistically significant and high fold-change regions.

- **D) Hierarchical Clustering Tree:** Samples are connected in a tree based on molecul similarity. Nodes are colored/labeled by group (C or NASH). Control and NASH samples form distinct subclusters, reinforcing that selected features drive strong group-level separation.
- **E) Classification Metrics Table:** Summarizes the final model's performance using common metrics for classifiers (ranging between 0 and 1). The accuracy of 0.99 means that in 99% of all classification tasks, the classifier could correctly assign a sample to its respective group. Lower accuracy would respectively mean a lower fraction of correctly classified samples.

All generated plots and tables containing the selected molecules can be downloaded after finishing step 5. The download folder includes a "readme.txt" file which explains the content of each file in this folder.

7 Comparison of OMEx to Random Forests and OPLS-DA

OMEx has been compared to the results of other molecule selection methods (see manuscript section "Comparison of OMEx to other molecule selection methods"). As an extension to this comparison, we performed molecule selection and classification using Random Forests (RF)[9,10] and Orthogonal Partial Least Squares Discriminant Analysis (OPLS-DA)[11,12], two common machine learning methods. Both analyses were performed in RStudio using R 4.5.1, using the ranger package (version 0.17.0)[10] and caret (version 7.0-1)[13] for RF analysis. For OPLS-DA, the ropls package (version 1.40.0)[12] was used.

Both methods were trained using a train-test split of 70%/30%, using random sampling to assign samples into training or test groups. After training on all molecules in the datasets, feature importance scores were extracted from the RF method directly and using variable influence on projection (VIP)[14] scores for OPLS-DA. Classification metrics were determined using the test set for the trained classifiers. Both workflows were repeated 100 times, each time with a new random split of the training and test datasets. The R scripts for both methods ("simpleOPLSDA.R" and "simpleRF.R") are included in the supplementary data ("benchmarking_results.zip").

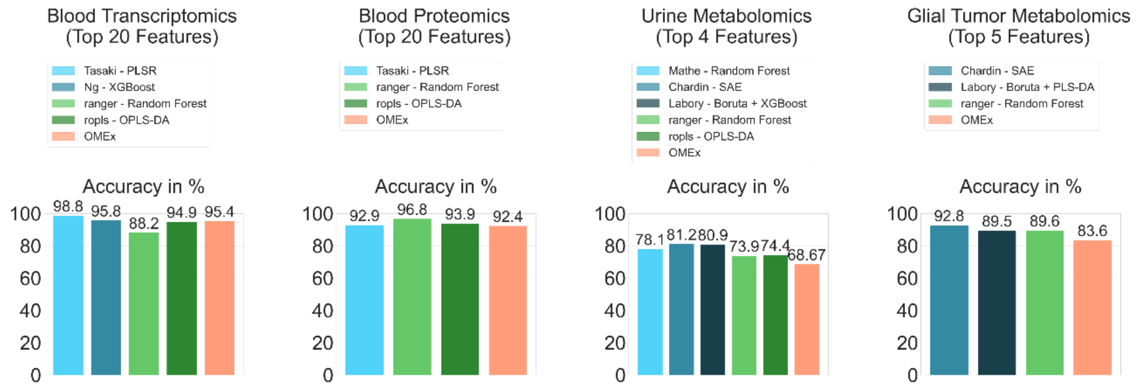

Figure S11: Performance comparison of all molecule selection/classification methods.

For the Glial Tumor Metabolomics dataset, OPLS-DA could not be performed, as the first predictive component was not significant (error message: "No model was built because the first predictive component was already not significant.").

The classification accuracies show that both Random Forest (RF) and OPLS-DA were able to separate most samples across datasets (Figure S11), performing within a 6% range of OMEx's accuracy. This is consistent with the results discussed in the main manuscript. However, unlike OMEx, neither RF nor OPLS-DA provides rankings or selections of potential molecule panels, which remains a key advantage of OMEx.

To compare molecule selection across methods, we visualized the top n molecules using upset plots (Figures S12–15). The number of top molecules was limited to the smallest number reported among all methods. For example, Chardin[7] reported the top 5 molecules, while Mathé[6] reported the top 4 for the urine and glial tumor metabolomics datasets, respectively. The upset plots illustrate the intersections of top n molecules identified by each method, similar to Venn diagrams. The intersection matrix at the bottom of each plot lists methods as rows and combinations of overlapping molecules as columns (indicated by black circles and connecting lines). The bar plot above each upset plot shows the size of these intersections.

Across all datasets, there is a consistent overlap of selected molecules between OMEx and other methods, with at least one molecule shared across all approaches. Each method, including OMEx, also identifies unique molecules. An exception is the glial tumor metabolomics dataset, where OMEx only shares overlapping molecules with the Boruta + PLS-DA method. However, in this case, the potential for overlap was limited by the comparison of only the top 5 molecules. These results support the conclusion that OMEx reliably identifies key molecules, including both shared and method-specific (unique) molecules.

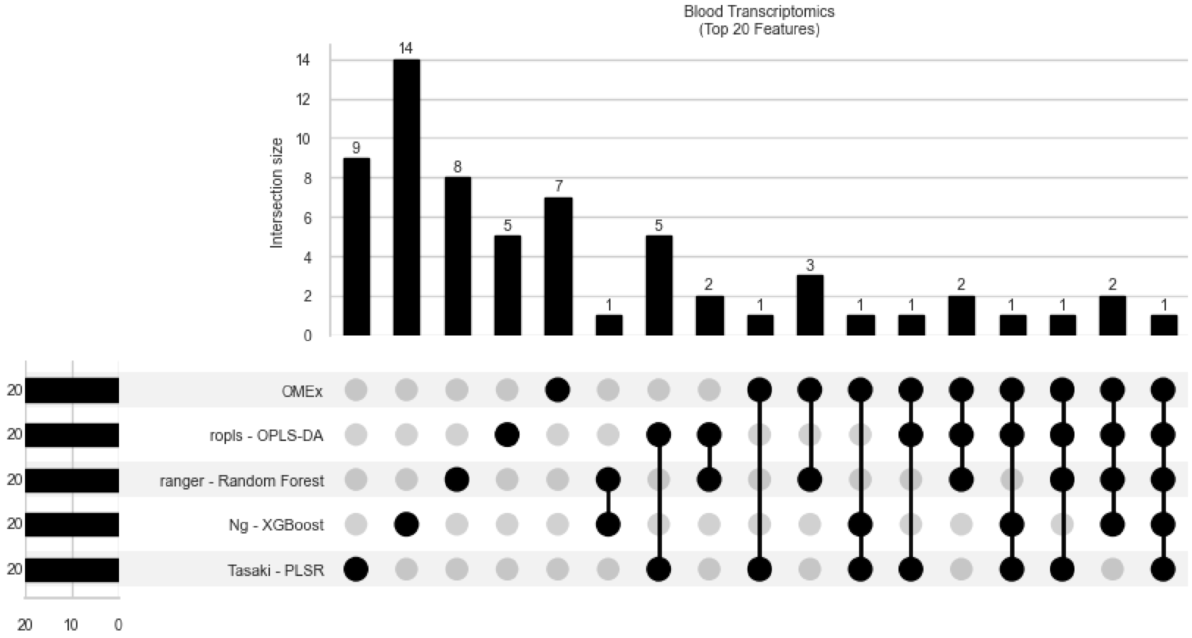

Figure S12: Intersections of top 20 molecules for the blood transcriptomics dataset.

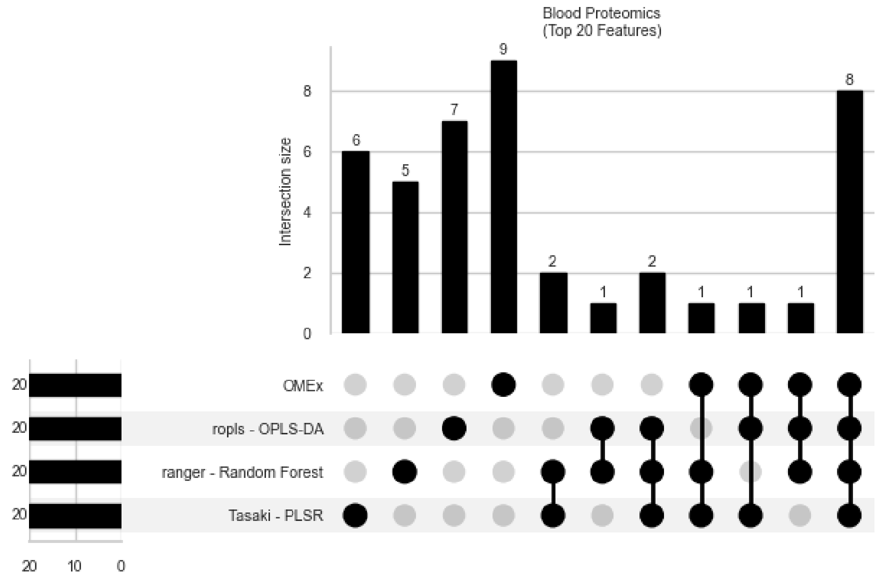

Figure S13: Intersections of top 20 molecules for the blood proteomics dataset.

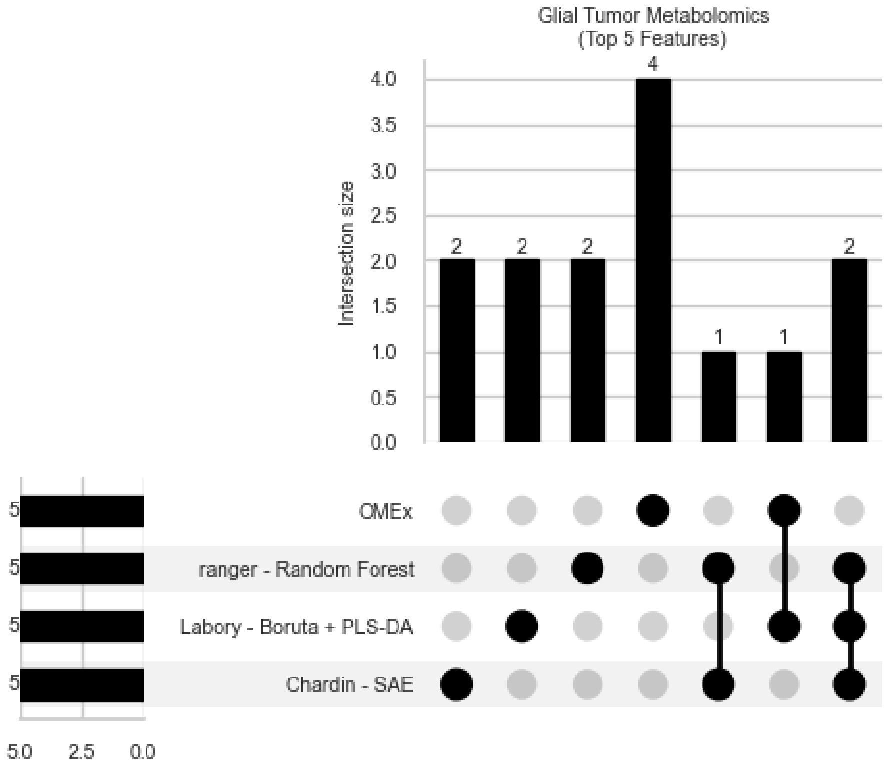

Figure S14: Intersections of top 20 molecules for the glial tumor metabolomics dataset.

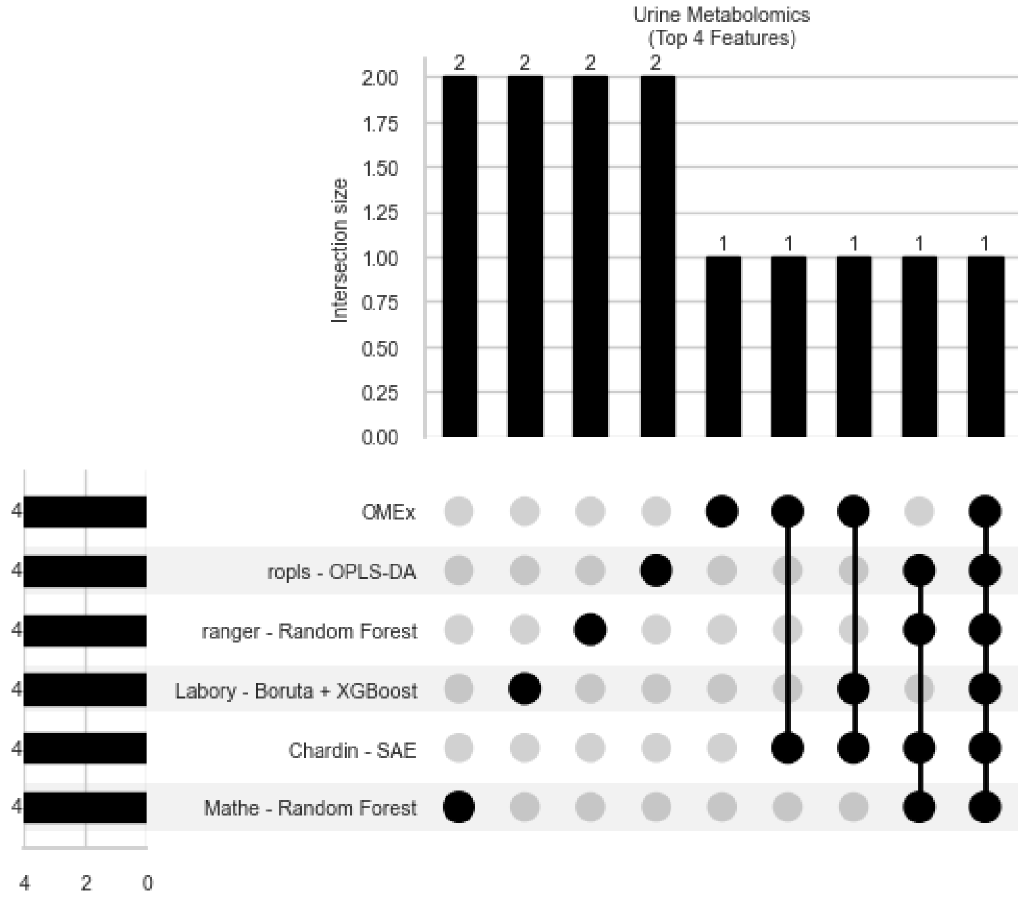

Figure S15: Intersections of top 20 molecules for the urine metabolomics dataset.

8 Generalization of molecules found by OMEx

The validation of potential biomarkers identified in a single dataset typically requires extensive laboratory work, including the collection of new samples from independent patient cohorts, measurements on different devices, and clinical trials[15]. While such experimental validation is beyond the scope of this manuscript, we evaluated whether molecule panels identified by OMEx generalize to independent patient cohorts.

To this end, we reproduced a study by Reinders *et al.*, in which the authors identified a panel of eight proteins for classifying diffuse large B-cell lymphoma (DLBCL) into activated B-cell (ABC)-like and germinal center B-cell (GCB)-like subtypes[4]. The authors kindly provided the proteomics data used in their study, consisting of two independently measured cohorts: one for training (41 samples, 1862 proteins) and one for testing (41 samples, 1828 proteins). Samples were originally labeled as ABC, GCB, or unclassified based on gene expression profiling.

For our analysis with OMEx, we included only ABC and GCB samples, excluding unclassified samples. Additionally, samples MPI-846 and 83516 were removed due to missing labels or duplicate measurements. The training and test cohorts were merged into a single CSV file and submitted to OMEx. The workflow was run with default parameters (table S5), except that the independent test set option was enabled, and the number of wrapper repetitions was increased to 5000.

To ensure evaluation was performed specifically on the independent test cohort, we manually updated OMEx's configuration file (config.json) after the initial workflow step, specifying the test sample IDs under responseData.overviewResponse.testSamples. Furthermore, the backend code for

the classification step (ClassifierJob.java) was modified to report classification metrics separately for the training set (via cross-validation) and the test cohort (based on the final trained classifier).

The two best molecule panels determined by OMEx were selected resulting in four molecules used for classification (table S6). There was no overlap between the panels reported by Reinder *et al.* and OMEx. The classification performance between both studies and datasets was comparable (table S7), demonstrating that the panel found by OMEx did generalize to the test cohort.

Table S6: Selected molecules for the study by Reinders et al.

method	Molecule panel
Lasso Regression (Reinders *et al.*)	P04233 | HG2A_HUMAN, Q15063 | POSTN_HUMAN, P51884 | LUM_HUMAN, Q9NS69 | TOM22_HUMAN, P62841 | RPS15_HUMAN, P16070 | CD44_HUMAN, P01871 | IGHM_HUMAN, P18031 | PTN1_HUMAN
OMEx	O95861|BPNT1_HUMAN, O43169|CYB5B_HUMAN, Q8IUX7|AEBP1_HUMAN, P22352|GPX3_HUMAN

Table S7: Classifier performance on the training and testing cohorts.

method	Training cohort accuracy	Test cohort accuracy
Lasso Regression (Reinders *et al.*)	82%	83.3%
OMEx	81% (cross validation)	86.7%

9 Installing OMEx locally using Docker Desktop

1. **Install Docker Desktop**: Download and install Docker Desktop from Docker's official website ([Docker Desktop: The #1 Containerization Tool for Developers | Docker]).
2. Open **Docker Desktop** on your machine.
3. Pull Docker Images
    a. In Docker Desktop, click on the "Images" tab in the left sidebar.
    b. Enter “mpacloud/omex-website-standalone” into the search input at the very top, click on the Docker image of that name and click on “Pull” (Tag: latest). This will download the Docker image of OMEx’s frontend.
    c. Repeat for OMEx’s backend (image “mpacloud/omex-server-standalone”).
4. Click the “images” tab on the left to view the list of your pulled images:

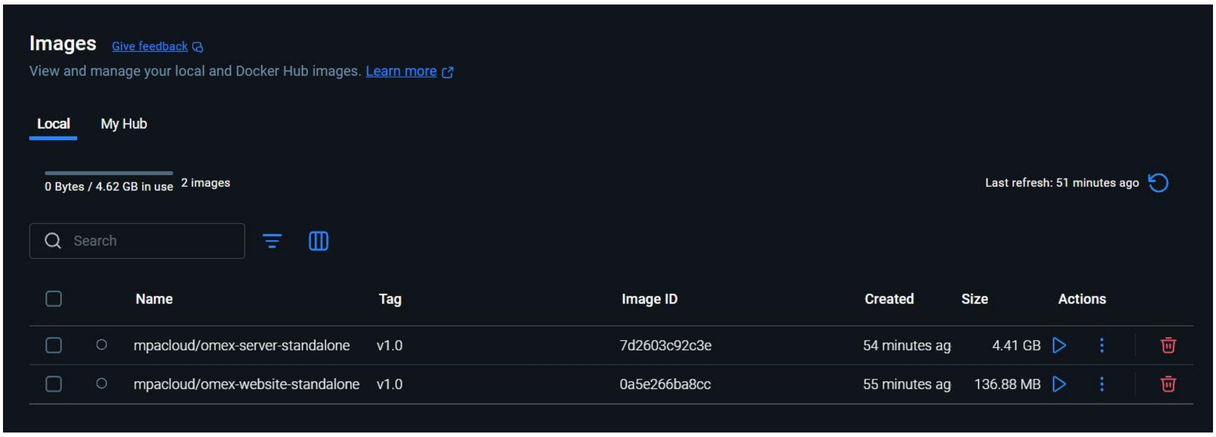

5. Click on the "play" button for the server and open the "optional settings" in the menu that has opened. Enter "9104" into the "Ports" input and click "run".

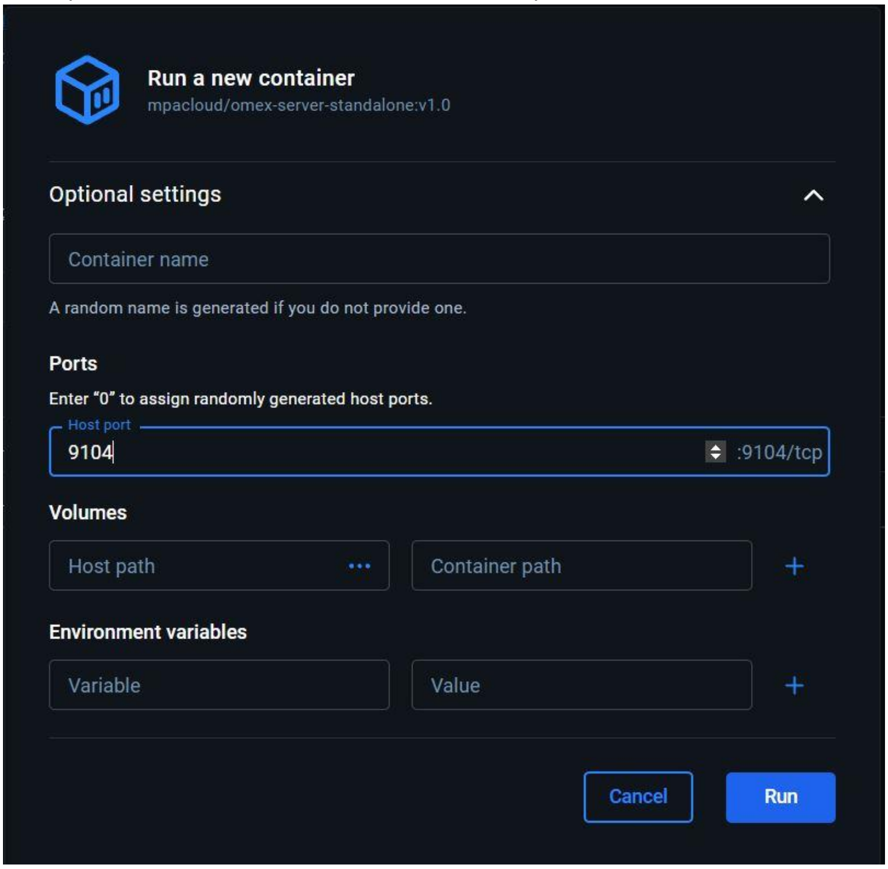

6. Click on the "play" button for the website and open the "optional settings" in the menu that has opened. Enter "9000" into the "Ports" (or any other) input and click "run".
7. Both containers should be running now. Click on the "containers" tab to verify.
8. Open your browser and visit the url "localhost:9000/omex" (use the port you specified for the website):

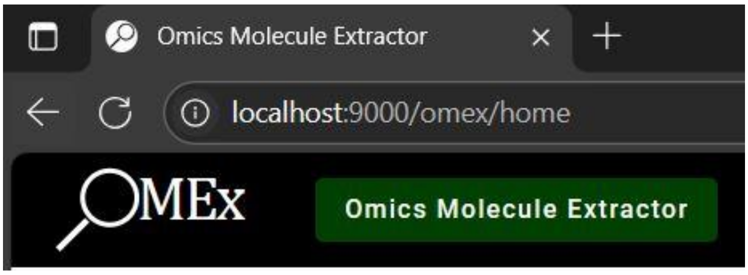

9. You can now use OMEx locally.

References

(1) Sydor, S.; Dandyk, C.; Schwerdt, J.; Manka, P.; Benndorf, D.; Lehmann, T.; Schallert, K.; Wolf, M.; Reichl, U.; Canbay, A.; et al. Discovering Biomarkers for Non-Alcoholic Steatohepatitis Patients with and without Hepatocellular Carcinoma Using Fecal Metaproteomics. *International Journal of Molecular Sciences* **2022**, *23* (16), 8841.

(2) Giacomoni, F.; Le Corguillé, G.; Monsoor, M.; Landi, M.; Pericard, P.; Pétéra, M.; Duperier, C.; Tremblay-Franco, M.; Martin, J.-F.; Jacob, D.; et al. Workflow4Metabolomics: a collaborative research infrastructure for computational metabolomics. *Bioinformatics* **2014**, *31* (9), 1493-1495.

(3) Bateman, A.; Martin, M.-J.; Orchard, S.; Magrane, M.; Adesina, A.; Ahmad, S.; Bowler-Barnett, E. H.; Bye-A-Jee, H.; Carpentier, D.; Denny, P.; et al. UniProt: the Universal Protein Knowledgebase in 2025. *Nucleic Acids Research* **2024**, *53* (D1), D609-D617.

(4) Reinders, J.; Altenbuchinger, M.; Limm, K.; Schwarzfischer, P.; Scheidt, T.; Strasser, L.; Richter, J.; Szczepanowski, M.; Huber, C. G.; Klapper, W.; et al. Platform independent protein-based cell-of-origin subtyping of diffuse large B-cell lymphoma in formalin-fixed paraffin-embedded tissue. *Scientific Reports* **2020**, *10* (1).

(5) Tasaki, S.; Suzuki, K.; Kassai, Y.; Takeshita, M.; Murota, A.; Kondo, Y.; Ando, T.; Nakayama, Y.; Okuzono, Y.; Takiguchi, M.; et al. Multi-omics monitoring of drug response in rheumatoid arthritis in pursuit of molecular remission. *Nature Communications* **2018**, *9* (1).

(6) Mathé, E. A.; Patterson, A. D.; Haznadar, M.; Manna, S. K.; Krausz, K. W.; Bowman, E. D.; Shields, P. G.; Idle, J. R.; Smith, P. B.; Anami, K.; et al. Noninvasive Urinary Metabolomic Profiling Identifies Diagnostic and Prognostic Markers in Lung Cancer. *Cancer Research* **2014**, *74* (12), 3259-3270.

(7) Chardin, D.; Gille, C.; Pourcher, T.; Humbert, O.; Barlaud, M. Learning a confidence score and the latent space of a new supervised autoencoder for diagnosis and prognosis in clinical metabolomic studies. *BMC Bioinformatics* **2022**, *23* (1).

(8) Ng, S.; Masarone, S.; Watson, D.; Barnes, M. R. The benefits and pitfalls of machine learning for biomarker discovery. *Cell and Tissue Research* **2023**, *394* (1), 17-31.

(9) Breiman, L. Random Forests. *Machine Learning* **2001**, *45* (1), 5-32.

(10) Wright, M. N.; Ziegler, A. ranger: A Fast Implementation of Random Forests for High Dimensional Data in C++ and R. *Journal of Statistical Software* **2017**, *77* (1).

(11) Trygg, J.; Wold, S. Orthogonal projections to latent structures (O-PLS). *Journal of Chemometrics* **2002**, *16* (3), 119-128.

(12) Thévenot, E. A.; Roux, A.; Xu, Y.; Ezan, E.; Junot, C. Analysis of the Human Adult Urinary Metabolome Variations with Age, Body Mass Index, and Gender by Implementing a Comprehensive Workflow for Univariate and OPLS Statistical Analyses. *Journal of Proteome Research* **2015**, *14* (8), 3322-3335.

(13) Kuhn, M. caret: Classification and Regression Training. 2007.

(14) Galindo-Prieto, B.; Eriksson, L.; Trygg, J. Variable influence on projection (VIP) for OPLS models and its applicability in multivariate time series analysis. *Chemometrics and Intelligent Laboratory Systems* **2015**, *146*, 297-304.

(15) Ou, F.-S.; Michiels, S.; Shyr, Y.; Adjei, A. A.; Oberg, A. L. Biomarker Discovery and Validation: Statistical Considerations. *Journal of Thoracic Oncology* **2021**, *16* (4), 537-545.
