# Supplementary material for: The Omics Molecule Extractor: A Web Application for the Selection of Potential Biomarker Panels
Source: J Proteome Res. 2025 Dec 1;25(1):484–90. doi: 10.1021/acs.jproteome.5c00176 (PMC12772118; doi:10.1021/acs.jproteome.5c00176)
Supplement: Supplementary file 1 [file pr5c00176_si_001.zip › supplementary_data/benchmarking_results/tasaki_ng_transcriptomics_cv/hacImage.html]

---
title: visNetwork
header-include: |

head: |2+
background-color: white
---
